# Exploring Dapagliflozin’s Influence on Autophagic Flux in Mania-like Behaviour: Insights from the LKB1/AMPK/LC3 Pathway in a Mouse Model

**DOI:** 10.1007/s11481-025-10218-1

**Published:** 2025-05-22

**Authors:** Nada K. Saleh, Sama M. Farrag, Mohamed F. El-Yamany, Ahmed S. Kamel

**Affiliations:** 1 Pharmacology and Toxicology Department, Faculty of Pharmacy, Cairo University, Cairo, 11562 Egypt; 2 Pharmacology and Toxicology Department, College of Pharmaceutical Sciences and Drug Manufacturing, Misr University for Science and Technology (MUST), Giza, Egypt

**Keywords:** Autophagic flux, Dapagliflozin, GABA, HPA axis, PSD, Mania-like behaviour

## Abstract

Mania-like episodes are neuropsychiatric disturbances associated with bipolar disorder (BD). Autophagic flux disturbance evolved as one of the molecular mechanisms implicated in mania. Recently, Dapagliflozin (DAPA) has corrected autophagic signaling in several neurological disorders. Yet, no endeavours examined the autophagic impact of DAPA in mania-like behaviours. This study aimed to investigate the effect of DAPA on disrupted autophagic pathways in a mouse model of mania-like behaviour. Mania-like behaviour was induced through paradoxical sleep deprivation (PSD) using the multiple-platform method for a duration of 36 h. Mice were divided into three groups, with DAPA (1 mg/kg/day, orally) administered for one week. Behavioural assessments were conducted on the 7th day. DAPA mitigated anxiety-like behaviour in the open field test and improved motor coordination and muscle tone in the rotarod test. Mechanistically, DAPA activated hippocampal autophagy-related markers; liver kinase B1/AM*P-*activated protein kinase (LKB1/AMPK) pathway, autophagy related gene 7 (ATG7), and microtubule-associated protein light chain 3II (LC3II). This was associated with reduced levels of the autophagosome receptor p62 protein, which subsequently enhanced GABA_A_ receptor-associated protein (GABARAP), facilitating the surface presentation of GABA_A_ receptors. Additionally, DAPA upregulated the GABA_B_ receptor R2 subunit through trophic factors such as brain-derived neurotrophic factor (BDNF) and glial cell line-derived neurotrophic factor (GDNF). Furthermore, DAPA mitigated elevated serum stress hormones and restored the balance between proinflammatory and anti-inflammatory cytokines in both cortical and hippocampal tissues. These findings highlight the role of autophagic flux modulation by DAPA and its therapeutic potential in mitigating mania-like behaviours.

**Graphical Abstract:**

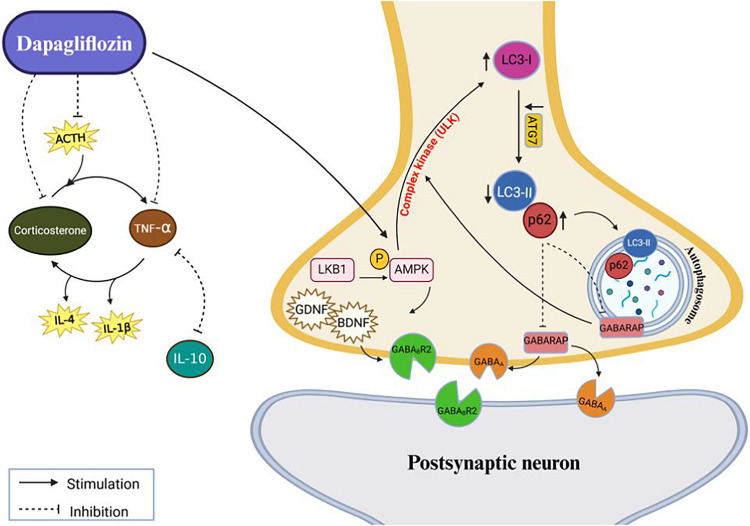

**Supplementary Information:**

The online version contains supplementary material available at 10.1007/s11481-025-10218-1.

## Introduction

The magnitude of bipolar disorder (BD) and its debilitating functional deficits comprise a huge social burden for its sufferers (Ferrari et al. 2016; Kebede et al. 2006). According to WHO, there are an estimated 40 million bipolar patients worldwide (https://www.who.int/news-room/fact-sheets/detail/bipolar-disorder). While the magnitude of the cortex and hippocampus in mood disorders is equivocal, studies emphasized prominent structural anomalies observed in the hippocampi of BD patients. The findings include decreased hippocampal volume, reduced hippocampal blood flow, cell density abnormalities, markers of decreased hippocampal neurogenesis/neuronal plasticity and cognitive deficits (Bearden et al. 2008; Cao et al., 2017; Chepenik et al. 2012). Generally, BD patients are fluctuated between alternating episodes of mania and depression, where mania is represented by irritability, decreased appetite/sleep, hallucinations, delusions and impulsivity (Jain & Mitra 2024). Notably, sleep disturbance, evolving as a consequence of mania, has been closely associated with mood dysregulation in BD. Interestingly, the relation between circadian rhythm and cellular autophagy was well-documented in numerous studies (Pastore et al. 2019; Wu et al. 2019)**.** Indeed, autophagic disturbance is well-recognized to negatively impact affective disorders including mania (Kato & Kato 2000; Yang et al. 2023)**.** This notion of attenuated autophagy was discovered in neuronal cell culture of patients diagnosed with BD (Sumitomo et al. 2018).

**Scheme 1:**
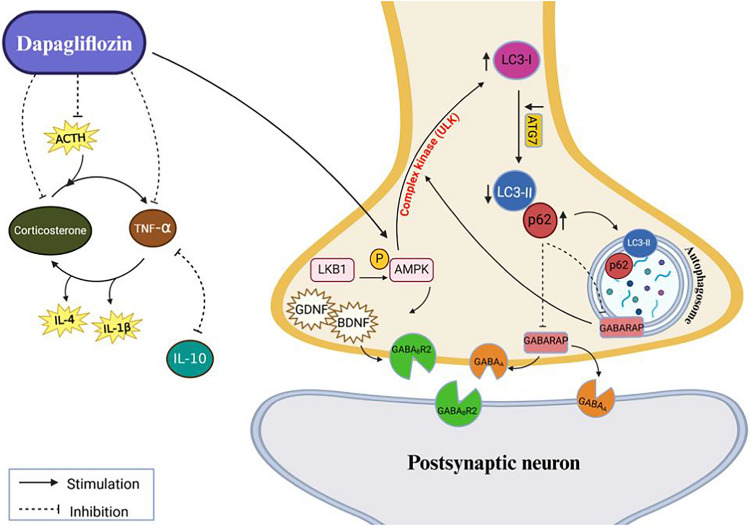
The activation of LKB1/AMPK/LC3 pathway by dapagliflozin is initiated by neurotrophic factors (BDNF & GDNF). AMPK activates serine/threonine protein kinase (ULK) enzymes responsible for a key step in autophagy; the conversion of LC3I to LC3II. This activation is completed by autophagosome formation and enhanced GABAergic transmission. Simultaneously, dapagliflozin mitigates stress hormones and subsequent inflammatory mediators. *ACTH: adrenocorticotropic hormone, AMPK: AMP-activated protein kinase, ATG7:autophagy related gene 7, BDNF: brain-derived neurotrophic factor, GABA*_*A*_*: gamma-aminobutyric acid receptor A, GABA*_*B*_* R2: gamma-aminobutyric acid receptor B subunit 2, GABARAP: GABA*_*A*_* receptor-associated protein, GDNF: glial cell line-derived neurotrophic factor, IL-1β: interleukin-1 beta, IL-4: interleukin-4, IL-10: interleukin-10, LC3I: microtubule-associated protein light chain 3I, LC3II: microtubule-associated protein light chain 3II, LKB1: liver kinase B1, p62: p62 protein, TNF-α: tumor necrosis factor alpha*

Recently, a growing body of evidence documented the correlation between autophagic dysfunction and manic disorders (Yang et al. 2023). Noteworthy, the genetic and proteomic expressions of autophagic effector proteins were suppressed in BD patients (Scaini et al. 2019)**.** To add up, rapamycin, a well-known autophagy enhancer, reduced mania-like aggression and reward-seeking behaviour in black Swiss mice as well as amphetamine-induced hyperactivity (Kara et al. 2018)**.** In addition, several studies proclaimed that first‐line anti-manic drugs regulate neuronal autophagy (Li et al. 2013; Nurnberger et al. 2000; Williams et al. 2002). The pathophysiology of mania extends to involve enhanced neuroinflammation coupled with curbed GABAergic and neurotrophic factors. These hallmarks are regulated and affected by autophagic flux as well. To further explain, clinical research on BD patients proved that autophagic deficiency is implicated in the downregulation of gamma aminobutyric acid (GABA) signaling which may contribute to the onset of neuropsychiatric disorders (Hui et al. 2019; Petty et al. 1993). Autophagy protects against cellular debris, a key player in neuroinflammation (Bai et al. 2024)**.** Also, it hinders NOD-like receptor protein 3 (NLRP3) inflammasome activation and its subsequent proinflammatory cytokines which appear to be significantly elevated in all stages of mania (Fujii et al. 2024; Huang & Lin 2007; Rowland et al. 2018)**.** Moreover, deficits in autophagy were associated with mitochondrial abnormalities, decline in synaptic integrity and the released neurotrophic factors, as noted in BD patients (Bar-Yosef et al. 2019)**.** Previous literature suggested that disturbed mitochondrial function in BD is potentially a result of impaired autophagy (Toker & Agam 2015)**.** Existing literature indicates that disturbances in hippocampal autophagy are particularly relevant to the mechanisms underlying mania among many neurological disorders (Li et al. 2018; Merenlender-Wagner et al. 2015; Zhang et al. 2016). That being said, owing to the limited therapeutic options for treating mania, it is worthwhile to address autophagic restoration theory as a promising approach to overcome these challenges (Scheme 1).

The present anti-manic drugs viz; valproic acid and carbamazepine, were reported to upregulate autophagy, enhance GABAergic activity and alleviate mitochondrial dysfunction in different animal models (Fu et al. 2010; Renna et al. 2010). By the same token, one of the promising drugs for relieving neural disorders via autophagy and GABAergic pathways is dapagliflozin (DAPA). The antidiabetic is well-tolerated and has a credited safety profile (Ptaszynska et al. 2014)**.** DAPA showed positive effects on neuroinflammation and enhancing both neurotrophic and growth factors (Chen et al. 2024; Da et al. 2023). Furthermore, DAPA was proven to amend mitochondrial (Sa-Nguanmoo et al. 2017) and autophagic dysfunction that in turn improved animals’ behaviour (El-Sahar et al. 2020)**.** In addition, stimulating autophagy via liver kinase B1/AM*P-*activated protein kinase (LKB1/AMPK) pathway coalesced with GABAergic enhancement was noted after DAPA use in multiple neurodegenerative disorders (Kamel et al. 2022, 2023)**.** Worthy to mention, studies also documented the pivotal role of DAPA in depressive-like behaviour, improving overall resilience and enhancing neuroplasticity (Dong et al. 2023; Muhammad et al. 2021). However, until now no endeavours were made regarding the role of DAPA in mania-like behaviours in preclinical studies. Hereby, the current study aims at screening the ability of DAPA in counteracting manic episodes in mice exposed to paradoxical sleep deprivation (PSD). PSD, which selectively deprives rapid eye movement (REM) sleep, has been extensively used to induce mania-like behaviour (Gessa et al. 1995)**.** In mice, PSD is viewed as a robust model for mania due to its ability to trigger manic episode hallmarks, including hyperactivity, heightened sexual behaviour and aggression. (Benedetti et al. 2008)**.** Moreover, PSD was associated with invigorating the proinflammatory cascades and cognitive impairment in mice (Valvassori et al. 2017; Zhu et al. 2012). The fact that animals subjected to PSD showed suppressed neurotrophic functions (Dal-Pont et al. 2019) makes it a rather intriguing epitome for the current study.

## Materials and Methods

### Ethics Statement

This study adhered to the guidelines outlined in the ‘Guide for the Care and Use of Laboratory Animals’ (NIH publication No.85–23, updated in 2011), as endorsed by the Research Ethics Committee of the Faculty of Pharmacy at Cairo University, Cairo, Egypt with permit number [PT-3016]. All measures were taken to minimize distress to the animals.

### Animals

The study utilized thirty male *C57BL/6 J mice* (12–14 weeks), each weighing 30–35 gm, obtained from Theodor Bilharz Research Institute, Imbaba, Giza, Egypt. The sample size was determined using prior studies as a reference and was validated using the G*Power software (version 3.1, Informer Technologies, Düsseldorf, Germany). The parameters for the power analysis were: an effect size of 0.6, an alpha level of 0.05, and a power of 0.8. The mice were housed under constant environmental conditions: temperature of 25 ± 2 ºC, humidity levels at 60 ± 10% and a 12-h light/dark cycle (lights on from 7:00 AM to 7:00 PM). These thirty mice were chosen after a pre-screening process to exclude those with motor abnormalities. During the acclimatization and study periods, the mice were housed in groups of five per cage in standard polycarbonate cages with dimensions of 40 cm × 25 cm × 15 cm. The cages were cleaned and bedding was replaced daily to ensure hygiene. They were provided with standard food pellets and water ad libitum.

### Drugs

DAPA was obtained from Liptis Pharmaceuticals (Liptis Pharmaceuticals, 6 th of October, Giza, Egypt) in its pure/raw form as dapagliflozin propanediol monohydrate powder for experimental use and was gradually dissolved in distilled water with the addition of one drop of 1% Tween 80 (Cat. No: 9005–65-6, Sigma-Aldrich, St. Louis, Missouri, USA) to enhance solubility. DAPA was administered in dose 1 mg/kg per oral for 7 days (Han et al. 2021; Millar et al. 2017). The study selected this dose based on previous study showing its neuroprotective and anti-neuroinflammatory effects in mice hippocampus. This dose was shown to reduce corticosterone levels, thereby mitigating SD-induced neurodisturbance (Millar et al. 2017). Additionally, the same dose showed promising enhancement of mitochondrial biogenesis and autophagy, key mechanisms implicated in mood regulation and neuroprotection in mice (Han et al. 2021). The dose was prepared as a solution and administered orally at a volume of 10 ml/kg body weight to ensure consistent dosing (Diehl et al. 2001; Turner et al. 2011a, b). The solution was delivered via oral gavage using blunt flexible polyethylene tubing to avoid any harms of esophageal perforation, aspiration, or mucosal trauma. The solution was administered slowly to prevent reflux, with monitoring for any signs of procedural compromise like coughing, choking, or resistance. A well-trained experimenter conducted all dosing procedures at a fixed time of day to ensure standardized systemic drug exposure.

### Induction of Mania by Paradoxical Sleeping Deprivation protocol (PSD)

**Scheme 2:**
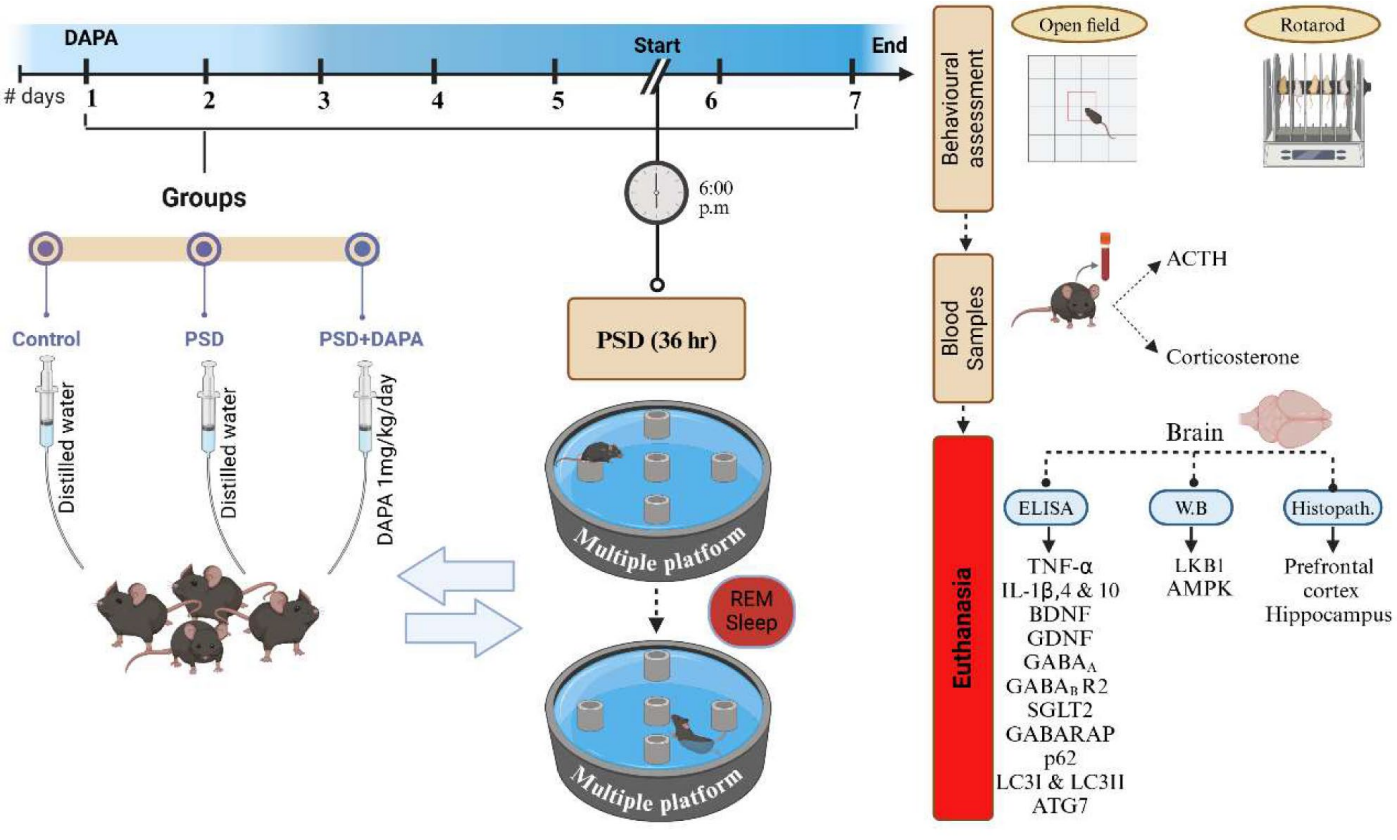
Outline of the study timeline and experimental design. *ACTH: adrenocorticotropic hormone, AMPK: AMP-activated protein kinase, ATG7:autophagy related gene 7, BDNF: brain-derived neurotrophic factor, DAPA: dapagliflozin, GABA*_*A*_*: gamma-aminobutyric acid receptor A, GABA*_*B*_* R2: gamma-aminobutyric acid receptor B subunit 2, GABARAP: GABA*_*A*_* receptor-associated protein, GDNF: glial cell line-derived neurotrophic factor, IL-1β: interleukin-1 beta, IL-4: interleukin-4, IL-10: interleukin-10, LC3I: microtubule-associated protein light chain 3I, LC3II: microtubule-associated protein light chain 3II, LKB1: liver kinase B1, p62: p62 protein, PSD: paradoxical sleep deprivation, PSD* + *DAPA: animals subjected to PSD treated with dapagliflozin, REM: rapid eye movement, TNF-α: tumor necrosis factor alpha*

Mice were subjected to multiple platform method for PSD induction, where REM phase is the sleep phase in which paradoxical sleep happens, is characterized by rapid eye movement and muscle atonia. Once mice start to enter REM phase, they tend to fall into the water and wake up immediately (Coenen & Van Hulzen 1980; Diao et al. 2023). Containers measuring 38 × 31 × 17 cm were equipped with five platforms (3.5 cm diameter). The containers were filled with water up to a depth of 1 cm. This setup compelled the animals to perch on the platforms, though they had the liberty to move between platforms. The setup contained two mice in the experiment to minimize psychosocial, immobilization, and separation stress. Once they transitioned into the paradoxical sleep stage and experienced muscle relaxation, they would fall into the water, waking them up. The method was applied for a 36-h PSD period. This induction method led to hyperactivity in mice, similar to mania. During this PSD phase, mice were briefly brought for treatments and promptly returned post-injection (Roybal et al. 2007; Tufik et al. 2009) (Scheme 2)**.**

### Experimental Design

Using a computer-generated table, mice were randomly assigned into three groups (*n* = 10/group). The first group received distilled water orally and served as home-caged controls where mice remained in their initial cages, with floors layered with sawdust for the duration of the experiment. The second group, received distilled water orally and on the fifth day, mice subjected to PSD protocol commenced at 6 p.m. for 36 h. The third group was administered DAPA (1 mg/kg, orally) for 7 days (Han et al. 2021; Millar et al. 2017) and undergone PSD protocol on the fifth day for 36 h till the seventh day (Valvassori et al. 2017). The 7-days treatment duration followed prior study evaluating hypothalamic–pituitary–adrenal (HPA) axis amendments in mania-like behaviour that included lithium as a reference (Valvassori et al. 2017). On the seventh day of the experiment, a series of behavioural assessments were executed, starting with the least to the most stressful tests as the following sequence: open field then rotarod tests. Researchers were completely blinded to group assignments during behavioural tests and data analysis to reduce observer bias. After these tests, *sera* were obtained from blood samples withdrawn from each mouse’s retro-orbital sinus under the influence of Pentobarbital sodium (Cat. No: 57–33-0, Sigma-Aldrich, St. Louis, Missouri, USA). Under anesthesia, the mice were humanely euthanized via decapitation (Dal-Pont et al. 2019; Pang & Laferriere 2020). The brains were isolated and divided into 2 subsets. The frontal cortex and hippocampus in the first subset (*n* = 7) were excised and flash-frozen in liquid nitrogen. To minimize freeze–thaw cycles, samples were aliquoted to use each aliquot only once to preserve protein integrity and stored at −80 °C. Samples were processed immediately after thawing to further reduce the risk of LC3-I degradation. Then, they were divided into 2 halves where the first half (*n* = 3) was utilized for biochemical analysis using western blot while second half (*n* = 6) was utilized for ELISA techniques. The second subset (*n* = 3) was prepared for histopathological examinations after fixing the whole brains in 10% buffered formol saline. During the samples’ analysis, the samples’ identities were unknown to all investigators. Another investigator was responsible for coding and decoding the samples.

#### Behavioural Tests

##### Open Field Test

The Open Field Test was conducted to assess the spontaneous movement of the animals. The apparatus was composed of a square wooden enclosure measuring 80 × 80 × 40 cm with white floor marked with black lines into 16 uniform squares. The test was performed under soft white lighting in a noise-free room, with an overhead camera capturing the movements of the animals. Each mouse was placed at the center of the enclosure, allowed to roam freely for a duration of 5 min, and then relocated to its original cage. After each session, the floor was cleaned. The recorded metrics were center and thigmotaxis time as well mean speed, center and total distances covered. The ANY-Maze video tracking system (Version 7.4, Stoelting Co., Wood Dale, Illinois, USA) was employed to record the animals’ movement (Roybal et al. 2007)**.**

##### Rotarod Test

The Rotarod test was conducted to assess the impact of mania-like behaviour on the animals’ muscle coordination and balance. The apparatus, measuring 120 cm in length and 3 cm in diameter, rotated consistently at 25 rpm. The apparatus consists of five equal chambers elevated 30 cm above a soft, padded cushion placed beneath it to avoid any harm from the fall. Each animal underwent five separate training trials, each lasting 3 min. Then the probe test was conducted for a maximum duration of 3 min, and the time taken for each animal to fall was estimated (Deacon 2013)**.**

#### Histological Assessment

Brain tissue specimens (*n* = 3/group) were fixed in 10% neutral buffered formalin for 72 h to ensure optimal preservation. Following fixation, tissues underwent dehydration through ascending grades of ethanol (70% for 1.5 h, 90% for 1.5 h, and absolute ethanol for 3 h), followed by clearing in xylene for 4 h. Cleared specimens were then impregnated in soft pure paraffin through three graded stages (each for 1 h) at 56 °C, subsequently embedded in paraffin wax at 58 °C, and oriented in blocks for sectioning. For histological examination, one paraffin block was prepared per animal, and from each block, a single sagittal Sect. (3–5 µm thick) was obtained using a rotary microtome to ensure anatomical precision. Sections were stained with Hematoxylin and Eosin (H&E), mounted in Dibutyl phthalate polystyrene xylene. Histopathological investigation was performed in a blinded manner to eliminate bias. One section per animal was analyzed, capturing 11–14 high-resolution micrographs per section using a full HD microscope camera, controlled by a Leica application module for tissue section analysis (Leica Microsystems GmbH, Wetzlar, Germany). Images were captured from six randomly selected, non-overlapping high-power fields. These standard procedures of samples’ processing were conducted according to Culling (2013).

#### Biochemical Assessment

##### Enzyme-linked Immunosorbent Assays

Using mice-specific enzyme-linked immunosorbent assay (ELISA) kits and according to the manufacturer’s instructions, the following parameters were measured; adrenocorticotropic hormone (ACTH) (MyBioSource, San Diego, USA, Cat. No: MBS2700344), Corticosterone (MyBiosource, San Diego, USA, Cat. No: MBS494312), brain-derived neurotrophic factor (BDNF) (MyBiosource, San Diego, USA, Cat. No: MBS355435), glial cell line-derived neurotrophic factor (GDNF) (MyBiosource, San Diego, USA, Cat. No: MBS2507522), interleukin-1 beta (IL-1β) (RayBiotech, Georgia, USA, Cat. No: ELM-IL1b-CL-1), interleukin-4 (IL-4) (ThermoFisher, IL, USA, Cat. No: BMS613), interleukin-10 (IL-10) (ThermoFisher, IL, USA, Cat. No: BMS614), tumor necrosis factor alpha (TNF-α) (RayBiotech, Georgia, USA, Cat. No: ELM-TNFa-CL-1), GABA_A_ receptor (MyBioSource, San Diego, USA, Cat. No: MBS9342109), GABA_B_ receptor R2 subunit (GABA_B_ R2) (MyBiosource, San Diego, USA, Cat. No: MBS9338634). GABA_A_ receptor-associated protein (GABARAP) (Abbexa, Cambridge, UK, Cat. No: abx524914), p62 protein (MyBiosource, San Diego, USA, Cat. No: MBS3806181), autophagy related gene 7 (ATG7) (MyBiosource, San Diego, USA, Cat. No: MBS2104232), microtubule-associated protein light chain 3I (LC3I) (MyBiosource, San Diego, USA, Cat. No: MBS3806505), microtubule-associated protein light chain 3II (LC3II) (MyBiosource, San Diego, USA, Cat. No: MBS3806182). The results of ACTH were expressed by pg/ml, corticosterone by ng/ml, SGLT2, GABA_B_ R2, GABARAP, p62 and ATG7 by ng/mg, while GABA_A_ by μmol/mg and LC3I, LC3II, BDNF, GDNF, TNF-α, IL-1β, IL-4 and IL-10 by pg/mg. The study used the kit’s standard curve to generate concentrations in pg/ml, μmol/m or ng/ml, which were then normalized by dividing by the tissue protein concentration (in mg/ml), yielding results expressed as pg/mg protein, μmol/mg or ng/mg protein.

##### Western Blotting

Hippocampal samples (*n* = 3/group) were processed using the TriFast protein extraction kit (Peqlab, VWR company, Vienna, Austria, Cat. No. 30-2010P), following the manufacturer’s instructions. Tissue homogenization was done in 1 ml TriFast per 50–100 mg tissue on ice using polytron homogenizer (POLYTRON PT 10–35, Kinematica, Luzern, Switzerland). The samples were shaken with 0.2 ml of chloroform per 1 ml of TriFast, vortexed for 15 s, and incubated at room temperature for 3 min then centrifuged for 5 min at 12,000 g for extraction. Samples were then precipitated with isopropanol, washed, placed into a boiling water bath for solubilization and centrifuged at 4 °C for 10 min at 10,000 g. For electrophoresis, 30 µg of proteins were mixed with loading buffer and heated at 95 °C for 8 min then separated by 12% sodium dodecyl sulfate (SDS) gel. Samples were then loaded with the stacking gel run at 75 V for 10 min, followed by a 125 V run for 60–90 min. To prepare the running buffer, 10 × stock solution was made (30.3 g Tris base, 114 g glycine and 10 g SDS in 1 L; 8.1–8.6 pH). Post-electrophoresis, the proteins were transferred to PVDF membranes and placed into a transfer buffer prepared from 100 ml stock solution (250 mM Trizma base, 192 mM glycine, 10% (v/v) methanol), 100 ml methanol and 800 ml distilled water. The membranes were then incubated at room temperature in a blocking solution to avoid non-specific binding of antibodies to the membrane, using 5% BSA in TBST (0.1% Tween-20 in Tris-buffered saline) for 1 h. Subsequently, they were stored overnight at 4◦C in a solution with primary antibodies, including (*P-*^Ser428^) LKB1 from ThermoFisher Scientific, IL, USA (Cat. No. PA5-36,858, at a 1:1000 dilution) and (*P-*^Thr172^) AMPK-α_1,2_ (Cat. No. PA5-37,821, at a 1:700 dilution). Membranes were washed at room temperature in TBST with 0.1% Tween-20 for 30–60 min. Membranes were then incubated for 1 h at room temperature in a solution containing HR*P-*conjugated secondary antibody (0.1–0.5 µg/ml). Membranes were rewashed for 30–60 min with blotting buffer five times. The housekeeping protein used in this experiment was β-actin. Following chemiluminescence using the enhanced chemiluminescence (ECL) fluorescence detection kit (GE HealthCare, Buckinghamshire, UK, Cat. No. RPN2108), gel documentation system (Geldoc-it, UVP, UK) was applied for data analysis using TotalLab analysis software (www.totallab.com, version (1.01.). Results were presented as relative protein expression levels in arbitrary units (AU), with normalization against β-actin protein expression.

#### Statistical Analysis

All data in the work were tested for normality and homogeneity of variance assumptions. Data were presented as the Mean ± S.D. The results were analyzed using the One-Way Analysis of Variance (ANOVA) test and was followed by Tukey’s multiple comparison test for all metrics. To ensure that all data met the normality criteria, Shapiro–Wilk test was applied. Pearson’s correlation analysis was performed to assess the relationship between behavioural outcomes and key molecular factors. The statistical evaluation was performed using the GraphPad Prism software (version 9, San Diego, CA, USA). A *p-*value of < 0.05 was set as the significance threshold for all the statistical tests. In all the measured parameters, the effect size partial Eta squared (*η2*), statistical significance (*p-*value), F-value and degree of freedom (df) were reported.

## Results

### Effect of DAPA On Muscle Coordination in Mice Subjected to PSD

**Fig. 1:**
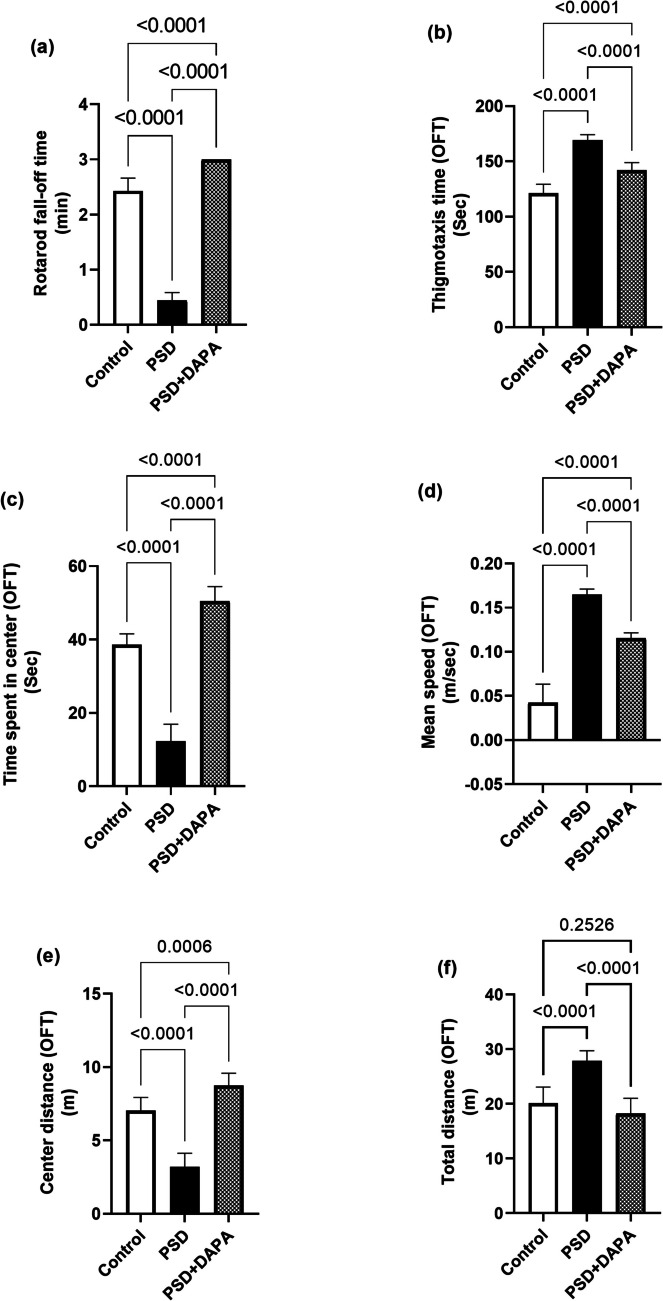
Impact of DAPA (1 mg/kg/day, p.o.) on behavioural performance in mice exposed to PSD. Each vertical line within each bar represents the mean ± S.D. (for each assessed parameter *n* = 10) (a) rotarod fall-off time, (b) thigmotaxis time, (c) time spent in center, (d) mean speed, (e) center distance and (f) total distance. Statistical analysis was performed by one-way ANOVA followed by Tukey’s post-hoc test, with the criterion for statistical significance: significant at *P* < 0.05. PSD: Paradoxical Sleeping Deprivation, DAPA: Dapagliflozin, OFT: open field test

The impact of PSD-induced muscular deficits and imbalance was confirmed by the rotarod test [F (2, 27) = 714.7, *P* < 0.0001, *η2* = 0.98]. As seen in Fig. 1a, the manic group showed a decrease in maximum time spent on the rotating rod by 82% compared to control group, while DAPA administration prolonged the time to reach 7 folds that of manic group.

### DAPA Enhanced the Motor Capabilities of Mania-induced Mice

PSD animals exhibited noticeable motor malfunctions which were documented by the open field test (for thigmotaxis time [F (2, 27) = 126.1, *P* < 0.0001, *η2* = 0.90], time spent in center [F (2, 27) = 250.7, *P* < 0.0001, *η2* = 0.95], mean speed [F (2, 27) = 223.7, *P* < 0.0001, *η2* = 0.94], center distance [F (2, 27) = 102.5, *P* < 0.0001, *η2* = 0.88] and total distance [F (2, 27) = 38.91, *P* < 0.0001, *η2* = 0.74]). Data presented in Fig. 1b, d and f) showed that model animals demonstrated an eminent increase in thigmotaxis time, mean speed and total distance by 39%, 287% and 39% respectively, compared to control animals. Meanwhile, Fig. 1c and e showed that time spent in center and the center distance by PSD animals were reduced by 68% and 55% respectively, compared to control group. Albeit, administration of DAPA boosted time spent in the center and center distance to reach 410% and 250% respectively, when compared to model animals. Interestingly, DAPA abridged thigmotaxis time, mean speed and total distance to reach 84%, 70% and 60%, respectively compared to PSD group.

### DAPA Mitigated the Inflammatory Outburst Induced By PSD

**Fig. 2:**
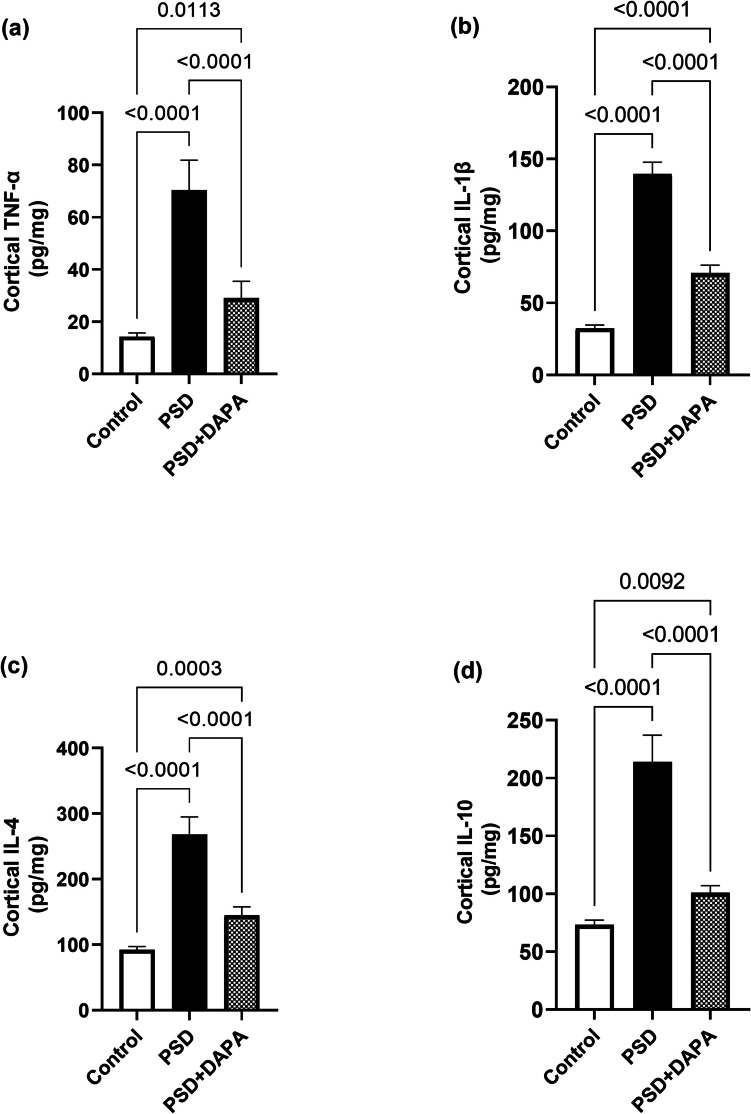
Influence of DAPA (1 mg/kg/day, p.o.) on inflammatory mediators’ level in the cortex of PSD mice. Each vertical line within each bar represents the mean ± S.D. (for each assessed parameter *n* = 6) (a) cortical TNF-α, (b) cortical IL-1β, (c) cortical IL-4, (d) cortical IL-10. Statistical analysis was performed by one-way ANOVA followed by Tukey’s post-hoc test, with the criterion for statistical significance: significant at *P* < 0.05. PSD: Paradoxical Sleeping Deprivation, DAPA: Dapagliflozin, TNF-α: tumor necrosis factor alpha, IL-1β: interleukin-1 beta, IL-4: interleukin-4, IL-10: interleukin-10

Alteration in the inflammatory mediators is an immune-mediated cascade presented in the prefrontal cortex and hippocampus of manic animals (for cortical TNF-α [F (2, 15) = 87.31, *P* < 0.0001, *η2* = 0.92], cortical IL-1β [F (2, 15) = 512.8, *P* < 0.0001, *η2* = 0.98], cortical IL-4 [F (2, 15) = 162.2, *P* < 0.0001, *η2* = 0.95] and cortical IL-10 [F (2, 15) = 171.5, *P* < 0.0001, *η2* = 0.96]). Data presented in Fig. 2 showed a major leap in cortical TNF-α, IL-1β, IL-4 and IL-10 in manic mice to reach 5, 5, 3 and 3 folds that of the control animals, respectively. In contrast, in DAPA mice, cortical TNF-α, IL-1β, IL-4 and IL-10 decreased to 41%, 50%, 54% and 47%, respectively of the manic group.

**Fig. 3:**
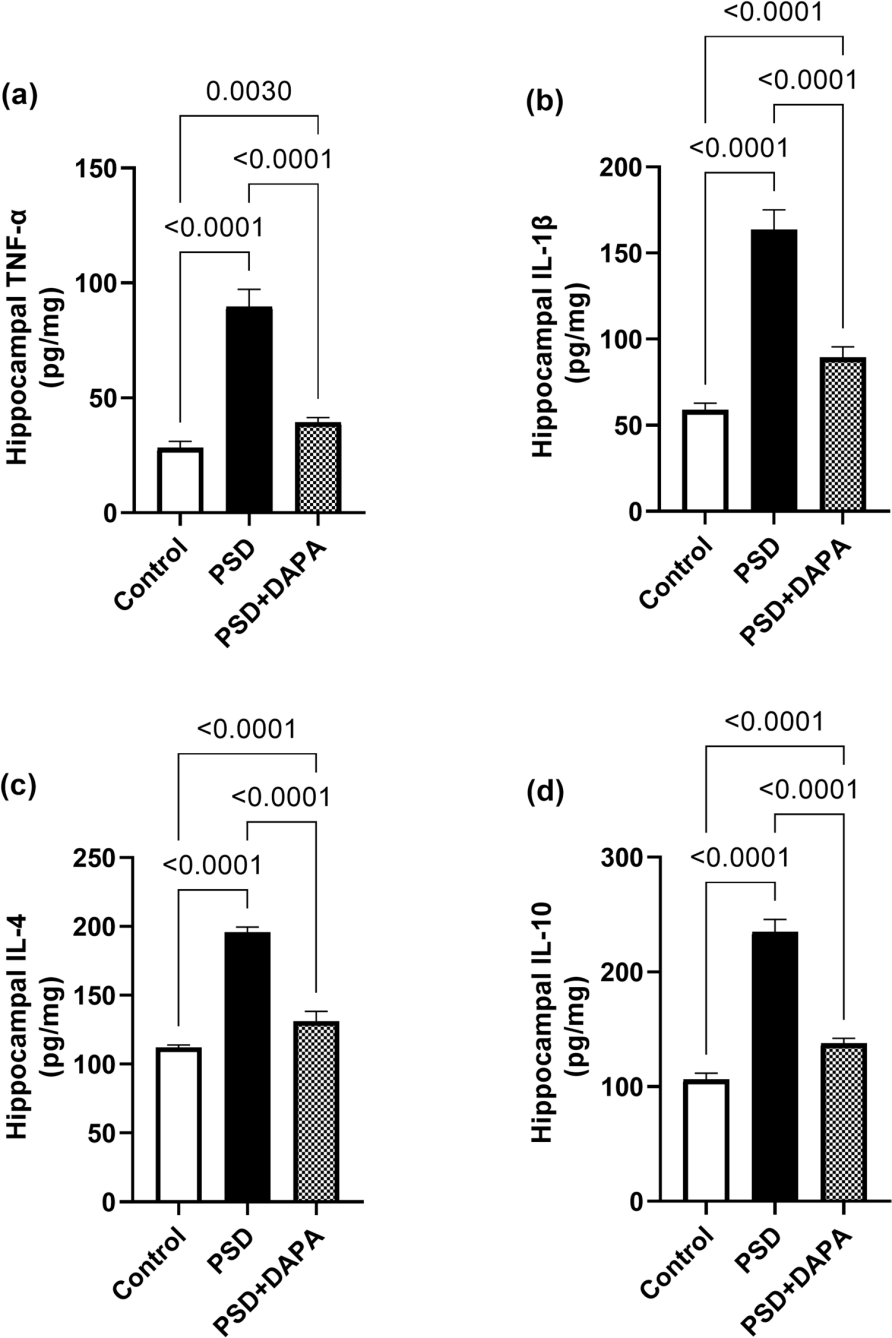
Effect of DAPA (1 mg/kg/day, p.o.) on inflammatory mediators’ levels in hippocampus of PSD mice. Each vertical line within each bar represents the mean ± S.D. (for each assessed parameter n = 6) (a) hippocampal TNF-α, (b) hippocampal IL-1β, (c) hippocampal IL-4, (d) hippocampal IL-10. Statistical analysis was performed by one-way ANOVA followed by Tukey’s post-hoc test, with the criterion for statistical significance: significant at P < 0.05. PSD: Paradoxical Sleeping Deprivation, DAPA: Dapagliflozin, TNF-α: tumor necrosis factor alpha, IL-1β: interleukin-1 beta, IL-4: interleukin-4, IL-10: interleukin-10

The same alterations were opted in hippocampus (for hippocampal TNF-α [F (2, 15) = 281.1, *P* < 0.0001, *η2* = 0.97], hippocampal IL-1β [F (2, 15) = 288.1, *P* < 0.0001, *η2* = 0.97], hippocampal IL-4 [F (2, 15) = 499.1, *P* < 0.0001, *η2* = 0.98] and hippocampal IL-10 [F (2, 15) = 503, *P* < 0.0001, *η2* = 0.98]). Meanwhile, hippocampal TNF-α, IL-1β, IL-4 and IL-10 appeared to increase in model animals to reach 3, 3, 2 and 3 folds the values in control group, respectively. Fortunately, DAPA administration reinstated the levels of TNF-α, IL-1β, IL-4 and IL-10 to 44%, 55%, 67% and 59%, respectively as seen in Fig. 3.

### DAPA Boosted Neurotrophic Factors’ Levels in Mania-induced Mice

**Fig. 4:**
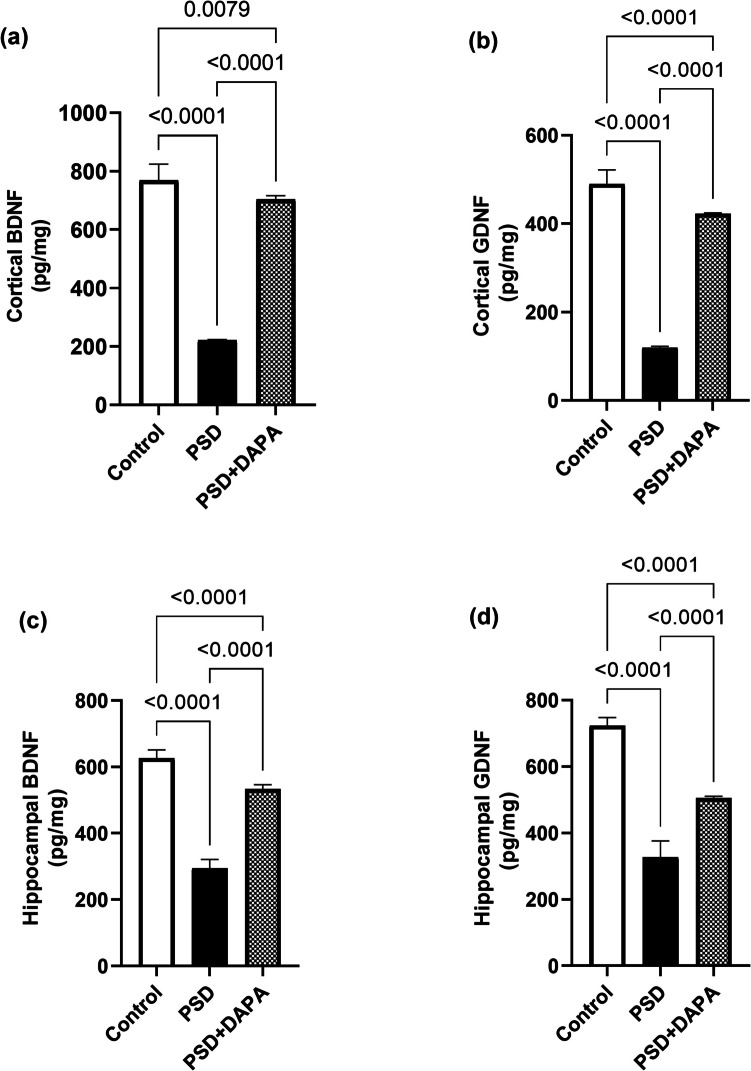
Consequences of DAPA (1 mg/kg/day, p.o.) on neurotrophic factors in mice exposed to PSD. Each vertical line within each bar represents the mean ± S.D. (for each assessed parameter *n* = 6) (a) cortical BDNF, (b) cortical GDNF, (c) hippocampal BDNF, (d) hippocampal GDNF. Statistical analysis was performed by one-way ANOVA followed by Tukey’s post-hoc test, with the criterion for statistical significance: significant at P < 0.05. PSD: Paradoxical Sleeping Deprivation, DAPA: Dapagliflozin, BDNF: brain-derived neurotrophic factor, GDNF: glial cell line-derived neurotrophic factor

Both BDNF and GDNF have been associated with neuronal survival and plasticity, especially during inflammatory flares (for cortical BDNF [F (2, 15) = 519.4, *P* < 0.0001, *η2* = 0.98] and cortical GDNF [F (2, 15) = 684.8, *P* < 0.0001, *η2* = 0.98]). However, these neurotrophic factors encountered fluctuation in their level in manic mice as illustrated in Fig. 4. Cortical BDNF and GDNF were diminished by 71% and 75% in PSD group respectively, compared to control. However, pretreatment with DAPA succeeded in enhancing BDNF and GDNF levels to 3 and 4 folds that of PSD group, respectively.

Similarly, manic mice showed a decline in hippocampal BDNF and GDNF by 53% and 55% respectively, compared to control mice, while DAPA augmented the level of these factors, both reaching 2 folds the manic group (for hippocampal BDNF [F (2, 15) = 350.1, *P* < 0.0001, *η2* = 0.97] and hippocampal GDNF [F (2, 15) = 234.8, *P* < 0.0001, *η2* = 0.96]).

### DAPA Restored Autophagy Sensors’ Disruption Evoked By PSD

**Fig. 5:**
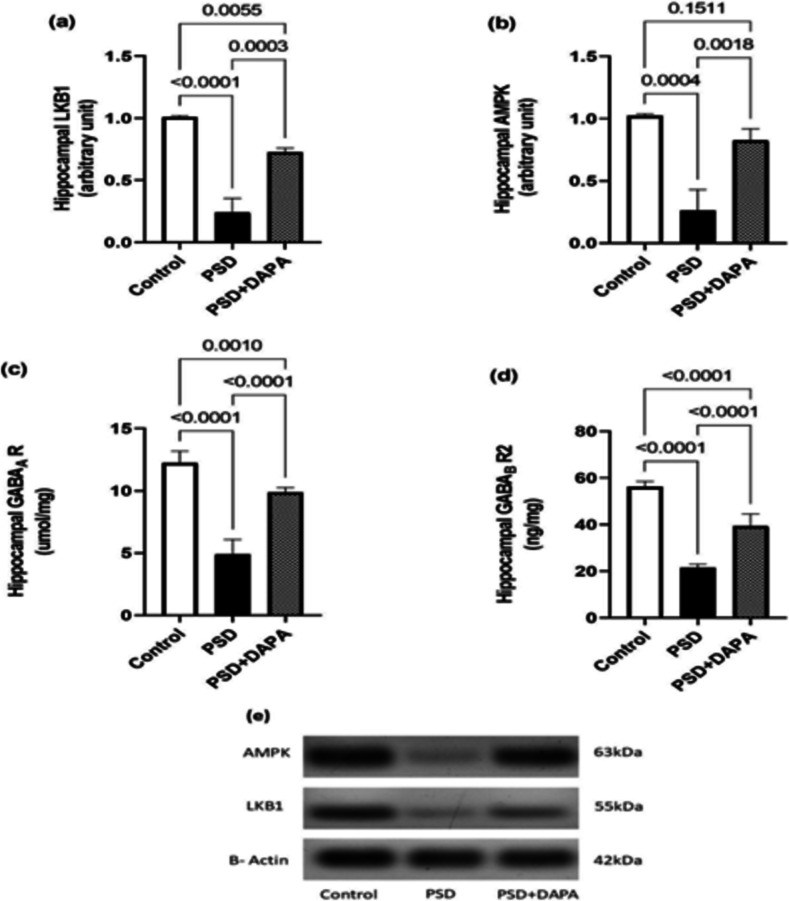
Influence of DAPA (1 mg/kg/day, p.o.) on autophagy sensors, GABA_A_ and GABA_B_ R2 receptors in mice exposed to PSD. Each vertical line within each bar represents the mean ± S.D (for LKB1 and AMPK; n = 3) while (for GABA_A_ R and GABA_B_ R2; n = 6). (a) hippocampal LKB1, (b) hippocampal AMPK, (c) hippocampal GABA_A_ R, (d) hippocampal GABA_B_ R2, (e) Imaging of western blots represent protein content of LKB1 and AMPK in mice hippocampus. Statistical analysis was performed by one-way ANOVA followed by Tukey’s post-hoc test, with the criterion for statistical significance: significant at P < 0.05. PSD: Paradoxical Sleeping Deprivation, DAPA: Dapagliflozin, LKB1: liver kinase B1, AMPK: AMP-activated protein kinase, GABA_A_ R: gamma-aminobutyric acid receptor A, GABA_B_ R2: gamma-aminobutyric acid receptor B subunit 2

Autophagy sensors, which are responsible for cellular metabolism and energy, are known to be regulated by multiple neurotrophic factors. Consequently, LKB1 and AMPK levels were disturbed in PSD animals (for hippocampal LKB1 [F (2, 6) = 97.27, *P* < 0.0001, *η2* = 0.97] and hippocampal AMPK [F (2, 6) = 38.79, *P* = 0.0004, *η2* = 0.92]). As seen in Fig. 5a and b. PSD mice showed a tremendous fall in both hippocampal LKB1 and AMPK by 74% and 76% respectively, compared to control animals. Meanwhile, PSD effect on LKB1 and AMPK was markedly reversed by DAPA revealing an upsurge to almost 3 folds the values of model group in both parameters.

### Impact of PSD and DAPA On GABA_A_ and GABA_B_ Receptors Levels

According to Fig. (5c and 5 d), the decline in both GABA_A_ and GABA_B_ R2 levels in PSD mice was by 60% and 62% respectively of the control group denoting a lack of the inhibitory machinery responsible for attenuating manic behaviour. Interestingly, DAPA administration led to a spike in the level of both GABAergic receptors to reach 2 folds that of model animals (for hippocampal GABA_A_ R [F (2, 15) = 105.7, *P* < 0.0001, *η2* = 0.93] and hippocampal GABA_B_ R2 [F (2, 15) = 159.9, *P* < 0.0001, *η2* = 0.95]).

### Impact of PSD and DAPA On Different Molecular Autophagy Markers

LKB1 and AMPK are not the only autophagy markers of concern in the present study, but also GABARAP, p62, ATG7, LC3I and LC3II were proven to have an important role in autophagosome functions and reflect any autophagy alteration (for GABARAP [F (2, 15) = 74.1, *P* < 0.0001, *η2* = 0.90], ATG7 [F (2, 15) = 295.1, *P* < 0.0001, *η2* = 0.97], LC3II [F (2, 15) = 78.08, *P* < 0.0001, *η2* = 0.97], LC3I [F (2, 15) = 76.83, *P* < 0.0001, *η2* = 0.91] and p62 [F (2, 15) = 291, *P* < 0.0001, *η2* = 0.91]).

**Fig. 6:**
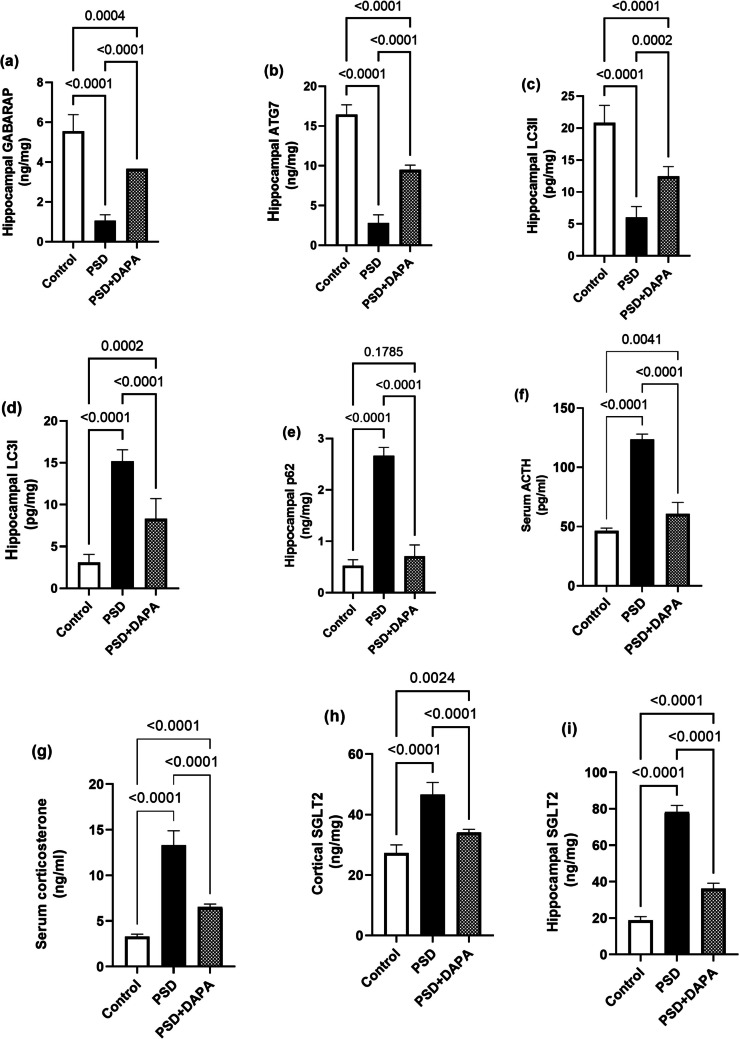
Effect of PSD and DAPA (1 mg/kg/day, p.o.) on hippocampal autophagy markers, on stress hormones and SGLT2 receptors in mice exposed to PSD. Each vertical line within each bar represents the mean ± S.D. (for each assessed parameter *n* = 6) (a) GABARAP, (b) ATG7, (c) LC3II, (d) LC3I, (e) p62, (f) serum ACTH, (g) serum corticosterone, (h) cortical SGLT2, (i) hippocampal SGLT2. Statistical analysis was performed by one-way ANOVA followed by Tukey’s post-hoc test, with the criterion for statistical significance: significant at P < 0.05. PSD: Paradoxical Sleeping Deprivation, DAPA: Dapagliflozin, GABARAP: gamma-aminobutyric acid receptor-associated protein, ATG7: autophagy related gene 7, LC3I/II: microtubule-associated protein light chain 3I/II, p62: autophagosome receptor p62 protein, ACTH: adrenocorticotropic hormone, SGLT2: sodium glucose-linked co-transporter 2

As seen in Fig. 6a-c. Hippocampal GABARAP, ATG7 and LC3II levels were diminished by 81%, 83% and 71% in manic group compared to control. Interestingly, DAPA increased their levels to 3, 3 and 2 folds respectively. While as demonstrated in Fig. (6 d and 6e). Hippocampal LC3I and p62 levels increased by 4 folds for both parameters in PSD group compared to the normal one, while they decreased in DAPA group to 55% and 27% respectively, compared to manic animals.

### DAPA Restored Serum ACTH and Corticosterone Levels

Owing to the reciprocal relationship between GABAergic receptors and stress hormones, alterations in ACTH and corticosterone were characteristic in manic mice (for serum ACTH [F (2, 15) = 246.3, *P* < 0.0001, *η2* = 0.97] and serum corticosterone [F (2, 15) = 179.7, *P* < 0.0001, *η2* = 0.95]). According to Fig. 6f and g, ACTH and corticosterone encountered a rise in PSD mice to 2 and 3 folds the values in normal mice, respectively. However, both ACTH and corticosterone showed a major decline by 49% upon treatment with DAPA compared to model animals.

### DAPA Attenuated SGLT2 Surge Induced By PSD

Biochemical analysis demonstrated in Fig. 6h and i, proved that the expression of SGLT2 both in the cortex and hippocampus of manic mice exhibited an augment to 2 and 4 folds the control group, respectively. Surprisingly, DAPA lessened SGLT2 expression to reach 73% and 46% in the cortex and hippocampus respectively, compared to PSD mice (for cortical SGLT2 [F (2, 15) = 70.9, *P* < 0.0001, *η2* = 0.90] and hippocampal SGLT2 [F (2, 15) = 639.2, *P* < 0.0001, *η2* = 0.98]).

### Correlation Test Assessment Between Each Molecular Factor and Behavioural Outcomes in the Rotarod and Open Field Tests

**Fig. 7:**
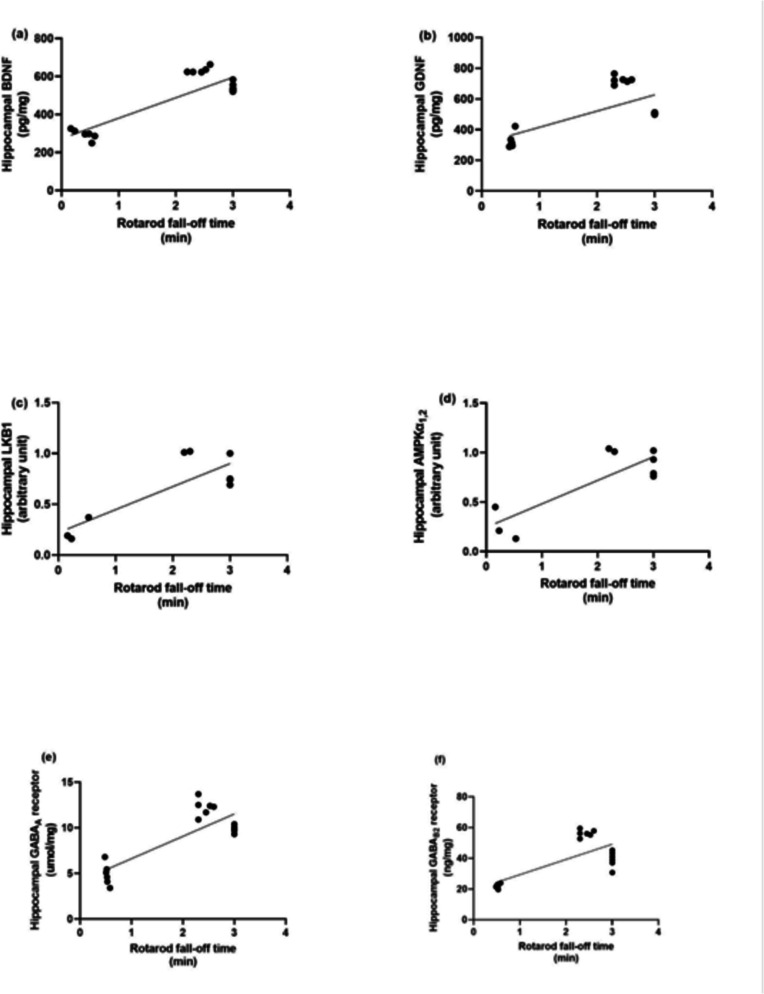
Linear regression model showing the correlation between rotarod fall-off time and (**a**) BDNF, (**b**) GDNF, (**c**) LKB1, (**d**) AMPK, (**e**) GABA_A_ and (**f**) GABA_B_ R2 using Pearson’s correlation, *P* < 0.05. BDNF: brain-derived neurotrophic factor, GDNF: glial cell line-derived neurotrophic factor, LKB1: liver kinase B1, AMPK: AMP-activated protein kinase, GABA_A_ R: gamma-aminobutyric acid receptor A, GABA_B_ R2: gamma-aminobutyric acid receptor B subunit 2

**Fig. 8:**
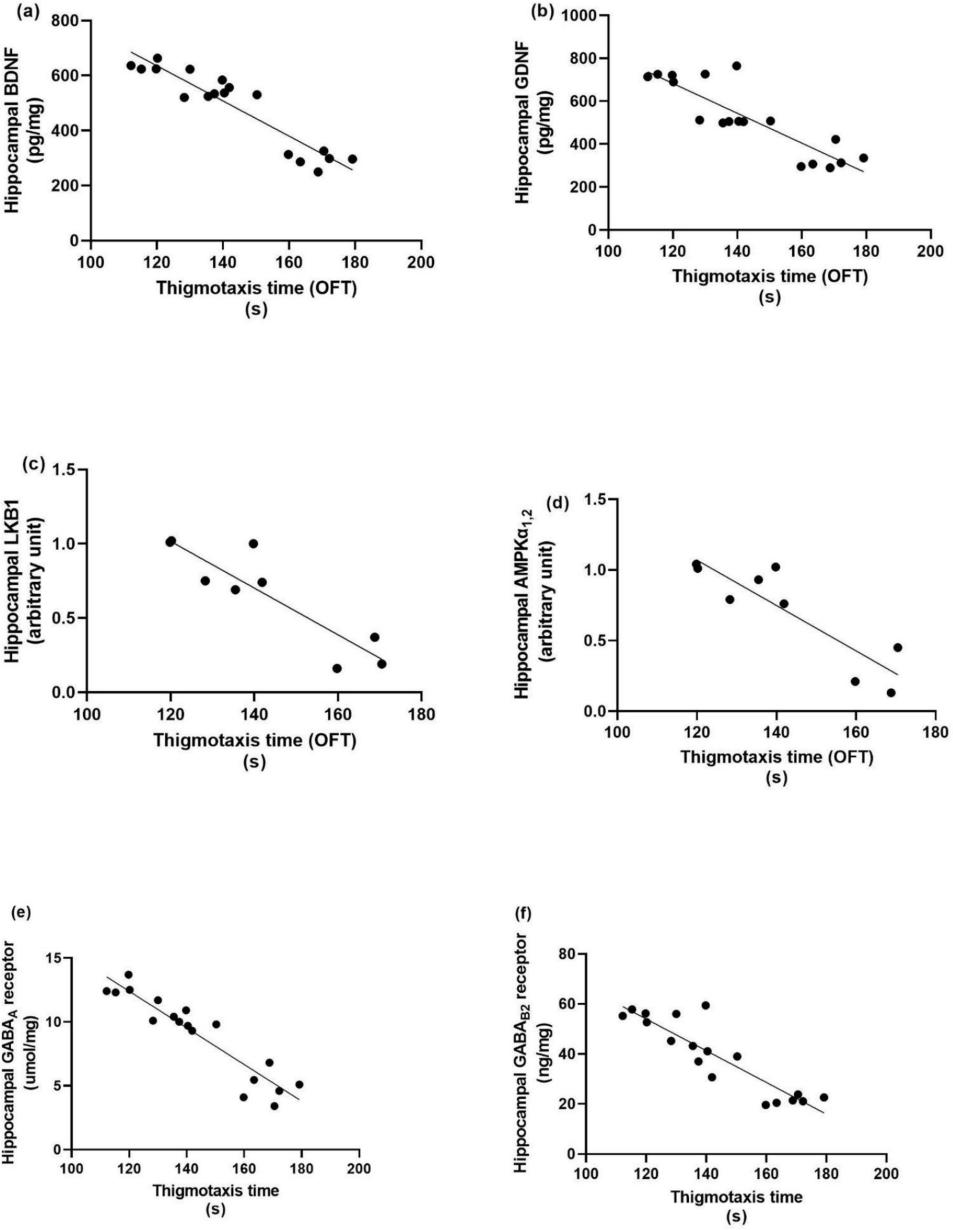
Linear regression model showing the correlation between thigmotaxis time and (**a**) BDNF, (**b**) GDNF, (**c**) LKB1, (**d**) AMPK, (**e**) GABA_A_ and (**f**) GABA_B_ R2 using Pearson’s correlation, *P* < 0.05. BDNF: brain-derived neurotrophic factor, GDNF: glial cell line-derived neurotrophic factor, LKB1: liver kinase B1, AMPK: AMP-activated protein kinase, GABA_A_ R: gamma-aminobutyric acid receptor A, GABA_B_ R2: gamma-aminobutyric acid receptor B subunit 2

**Fig. 9:**
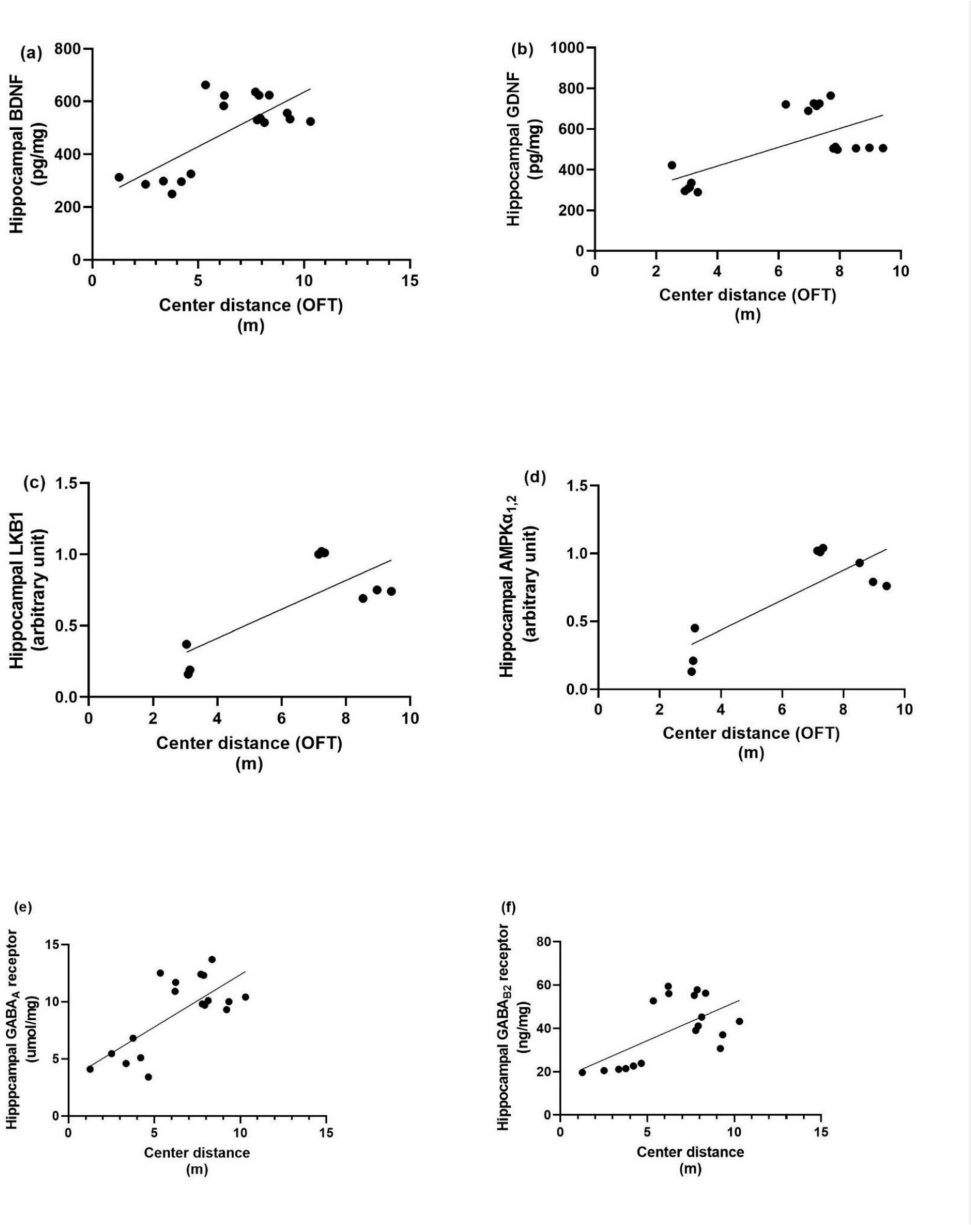
Linear regression model showing the correlation between center distance and (a) BDNF, (b) GDNF, (c) LKB1, (d) AMPK, (e) GABA_A_ and (f) GABA_B_ R2 using Pearson’s correlation, P < 0.05. BDNF: brain-derived neurotrophic factor, GDNF: glial cell line-derived neurotrophic factor, LKB1: liver kinase B1, AMPK: AMP-activated protein kinase, GABA_A_ R: gamma-aminobutyric acid receptor A, GABA_B_ R2: gamma-aminobutyric acid receptor B subunit 2

**Fig. 10:**
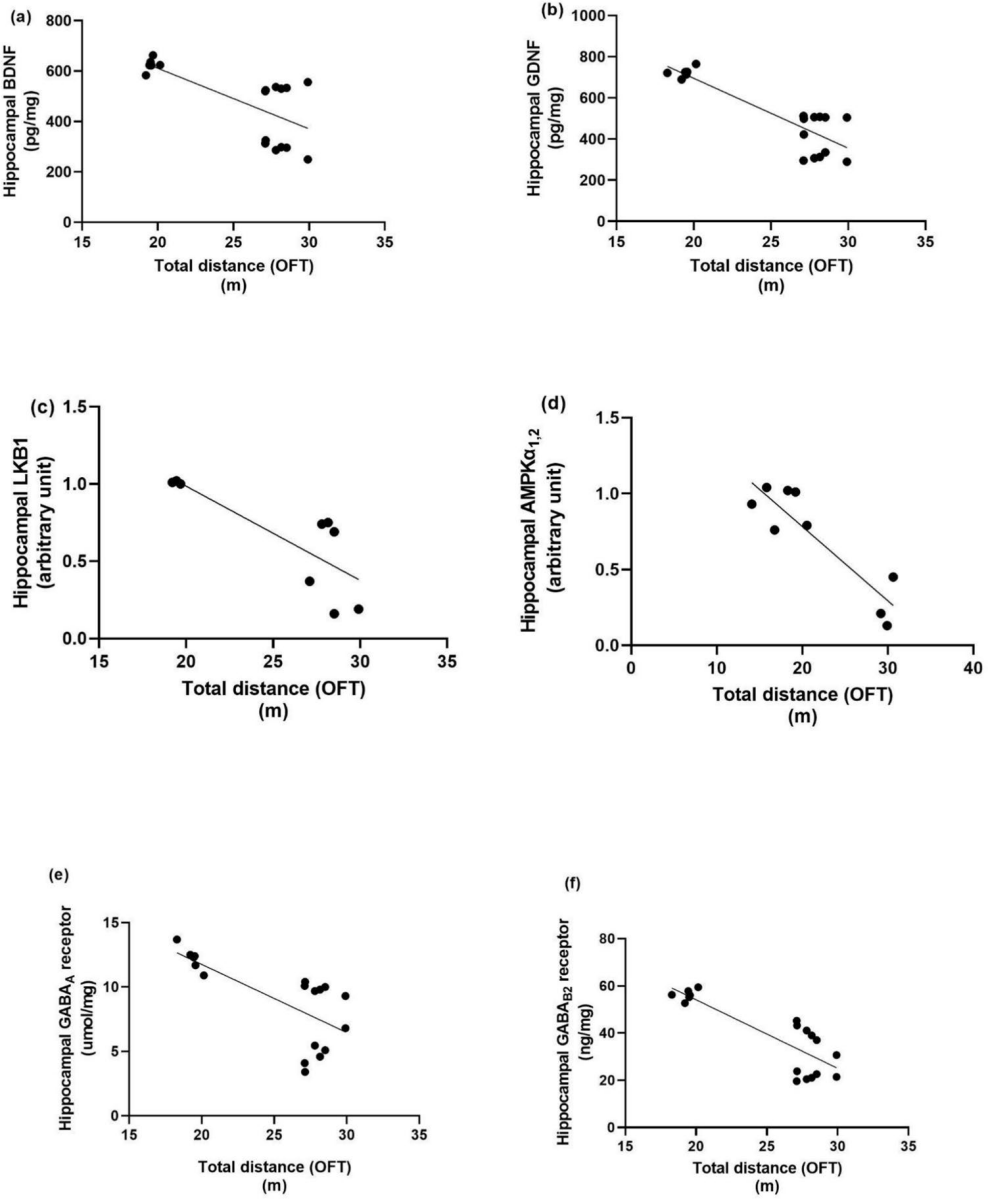
Linear regression model showing the correlation between total distance and (**a**) BDNF, (**b**) GDNF, (**c**) LKB1, (**d**) AMPK, (**e**) GABA_A_ and (**f**) GABA_B_ R2 using Pearson’s correlation, *P* < 0.05. BDNF: brain-derived neurotrophic factor, GDNF: glial cell line-derived neurotrophic factor, LKB1: liver kinase B1, AMPK: AMP-activated protein kinase, GABA_A_ R: gamma-aminobutyric acid receptor A, GABA_B_ R2: gamma-aminobutyric acid receptor B subunit 2

Linear regression model showing the correlation between anxiety-like behaviour, neurotrophic factors and autophagic markers as presented in Fig. 7, rotarod fall-off time is positively correlated with BDNF (*r* = 0.8435, *P* < 0.0001), GDNF (*r* = 0.6800, *P* < 0.0001), LKB1 (*r* = 0.8359, *p* = 0.005), AMPK (*r* = 0.8450, *p* = 0.0041), Hippocampal GABA_A_ R (*r* = 0.8183, *p* = 0.0019), Hippocampal GABA_B_ R2 (*r* = 0.7240, *p* = 0.0007). While as presented in Fig. 8 thigmotaxis time in open field test is negatively correlated with BDNF (*r* = −0.9320, *P* < 0.0001), GDNF (*r* = −0.8675, *P* < 0.0001), LKB1 (*r* = −0.8955, *p* = 0.0011), AMPK (*r* = −0.8834, *p* = 0.0016). Hippocampal GABA_A_ R(*r* = −0.9311, *P* < 0.0001), Hippocampal GABA_B_ R2 (*r* = −0.8983, *P* < 0.0001). As shown in Fig. 9, center distance is positively correlated with BDNF (*r* = 0.7361, *p* = 0.0005), GDNF (*r* = 0.6586, *p* = 0.003), LKB1 (*r* = 0.7754, *p* = 0.00141), AMPK (*r* = 0.8144, *p* = 0.0075), Hippocampal GABA_A_ R (*r* = 0.7259, *p* = 0.0006), Hippocampal GABA_B_ R2 (*r* = 0.6064, *p* = 0.0076). Figure 10 demonstrated that total distance in open field test is negatively correlated with BDNF (*r* = −0.6978, *p* = 0.0013), GDNF (*r* = −0.8676, *P* < 0.0001), LKB1 (*r* = −0.8012, *p* = 0.0094), AMPK (*r* = −0.8979, *p* = 0.001), Hippocampal GABA_A_ R (*r* = −0.7031, *P* < 0.0011),Hippocampal GABA_B_ R2 (*r* = −0.8425, *P* < 0.0001).

### Histopathological Amendments in DAPA Animals

**Fig. 11:**
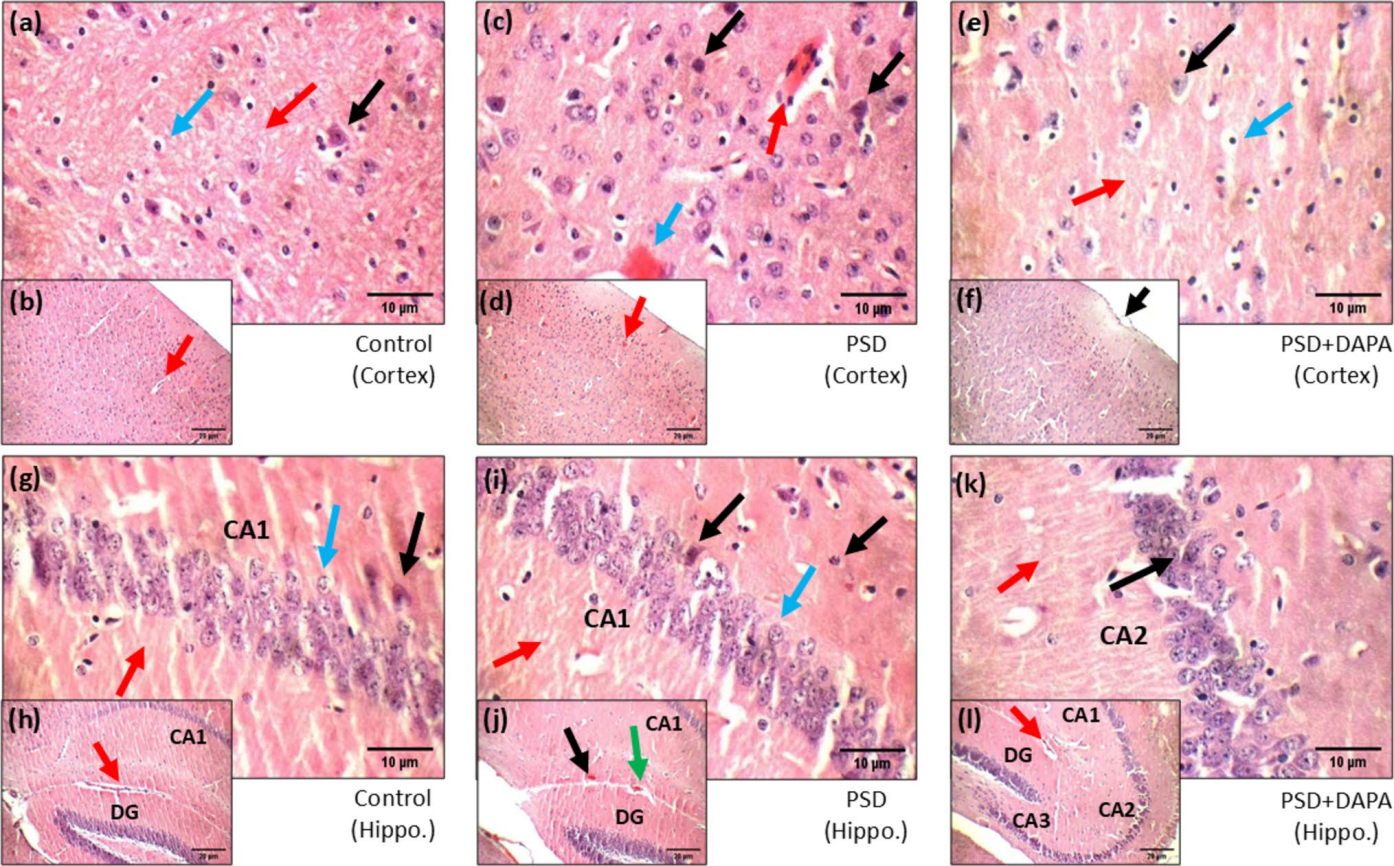
Effects of PSD and DAPA (1 mg/kg/day, p.o.) on histopathological sections of prefrontal cortex and hippocampal tissue of different experimental groups (*n* = 3) (200 × and 400 ×). Black, red, green and blue arrows in different sections demonstrate the alterations mentioned in the text. Sections were stained with hematoxylin and eosin (H&E) where (a&b): Control’s cortex, (c&d): PSD’s cortex, (e&f): PSD + DAPA’s cortex, (g&h): Control’s hippocampus, (i&j): PSD’s hippocampus and (k&l): PSD + DAPA’s hippocampus. Control: normal animals, PSD: animals subjected to PSD and PSD + DAPA: animals subjected to PSD treated with dapagliflozin, CA1-3: Cornu Ammonis (1–3), DG: Dentate gyrus

To emphasize the role of DAPA against neurodegeneration triggered by PSD, histopathological sections were inspected in both the cortex and the hippocampus. Microscopic examination of the prefrontal cortex in control group showed intact meninges with normal blood vessels (red arrow) (Fig. 11b), healthy neurons (black arrow) and normal average glial cells (blue arrow) in fibrillary background (red arrow) (Fig. 11a). Compared with control group, PSD animals were presented with degenerated neurons (black arrows) with eosinophilic plaque-like areas (red arrow) (Fig. 11d), while the white matter showed average glial cells in fibrillary background also with eosinophilic plaque-like areas (blue arrow) (Fig. 11c). Meanwhile, sections from DAPA-treated group showed completely well-structured meninges (black arrow) (Fig. 11f), intact neurons (black arrow), cerebral cortex with well-formed average glial cells (blue arrow) and normal fibrillary background (red arrow) (Fig. 11e).

On the other hand, histopathological examination of hippocampus in control mice showed normal blood vessels (red arrow) (Fig. 11h), average Cornu Amonis (CA1), normal dentate gyrus (DG) and intact pyramidal neurons (black arrow). Furthermore, well-organized granule cells (blue arrow) and well-proportioned inter-neuron area (red arrow) were observed (Fig. 11g). Contrarily, hippocampal sections of PSD mice showed scattered degenerated neurons (black arrow), average granule cells (blue arrow) and normal inter-neuron area (red arrow) (Fig. 11i), while congested blood vessels appeared in DG area (green arrow) (Fig. 11j) and eosinophilic plaque-like areas in CA1 (black arrow) (Fig. 11j). On the other hand, samples from DAPA-treated group showed well-preserved hippocampal structure with well-formed Cornu Amonis (CA1), (CA2) and (CA3), average dentate gyrus (DG), intact blood vessels (red arrow) (Fig. 11l), well-shaped pyramidal neurons (black arrow) surrounded by normal inter-neuron area (red arrow) (Fig. 11k).

## Discussion

In the current study, PSD was used to induce mania-like behaviour manifested by the distorted performance of the mice in both OFT and rotarod test. Several lines of evidence pointed out the negative effects of PSD on animals’ behaviour, most prominently, muscle imbalance and increased locomotive activity (Dal-Pont et al. 2019; Valvassori et al. 2017). That being said, DAPA was examined for its ability to control these manic episodes and reverse the hyperactivity as well as the muscle incoordination. The anti-diabetic drug succeeded in counteracting these alterations and improving animals’ performance in both behavioural tasks. These outcomes are in line with previous studies demonstrating DAPA’s ability to attenuate motor dysfunction in several neurodegenerative disorders (Arab et al. 2021; El-Sahar et al. 2020).

Slee*p-*deprived mice exhibit behavioural changes that closely resemble manic symptoms of BD, providing strong construct validity for this model. Evidence from human studies supports a link between sleep loss and the onset of mania, with reduced sleep serving as a reliable prodrome of manic episodes (Plante & Winkelman 2008). Sleep disruptions, especially following stress or changes in sleep schedules, have been shown as a risk factor for mania (Hensch et al. 2019). Sleep deprivation in experimental studies resulted in increased locomotor activity, a key feature of psychomotor agitation often associated with mania (Young et al. 2007). Hyperlocomotion is commonly used as a primary measure of manic-like behaviour in animal studies. In clinical settings, mania is characterized by increased energy levels, restlessness, and hyperactivity. Herein, the PSD mice showed significant increases in the speed and total distance covered in the OFT. This figures out the hyperlocomotion activity as observed in manic patients. However, mania encompasses a broader range of symptoms beyond hyperactivity. In this study, SD mice displayed additional behaviours aligned with manic symptoms, including heightened anxiety, which adds to the face validity of the model. In humans, experimental sleep deprivation showed prominent anxiety features (Mellman 2006). While the co-occurrence of anxiety and mania is still inconclusive, earlier studies depicted the high probability of anxiety as a comorbid event in manic patients (Das 2013; Kessler et al. 1996). Nevertheless, several rodent models displaying manic state were yet proven to manifest amplified anxiety-like behaviour (Bartsch et al. 2019; Weiss & Boss-Williams 2017). In fact, PSD animals exhibited higher levels of anxiety when compared to control ones, either following single/multiple platform methods (Silva et al. 2004a, b; Silva et al. 2004a, b) or intermittent/persistent SD (Yin et al. 2017). Moreover, previous reports linked heightened anxiety behaviour in SD animals to increased oxidative damage (Kumar & Singh 2009; Vollert et al. 2011). This conclusion is based on the fact that sleep acts as a repair mechanism, detoxifying the oxidative damage accumulated during the wake period (Ikeda et al. 2005). These findings are under debate as some studies claimed anxiolytic action during manic episodes generally (Shaltiel et al. 2008) and SD-induced mania specifically (Abbasi et al. 2024; Alvarenga et al. 2008). This dispute could be due to variations in species, duration of SD protocol and measures used in assessing anxiety behaviour. Notwithstanding, studies also pointed out that alterations in anxiety levels were associated with whether it was individual or grouped PSD (Suchecki et al. 2002). This aligns with a tendency of PSD animals to increase the time hanging walls; thigmotaxis more than the center of the arena. A well-documented finding in patients with BD and schizophrenia is the disruption of GABAergic neurons and associated markers in the hippocampus. Notably, the levels of GABA-synthesizing enzymes, GAD65 and GAD67, are significantly reduced in this brain region. This alteration may contribute to dysregulated inhibitory signaling, which is closely linked to heightened anxiety, as the hippocampus plays a critical role in modulating emotional responses and stress regulation (Wang et al. 2011). Besides, mRNA gene expression of GABA synthesizing enzymes was reduced in hippocampus of bipolar patients (Konradi et al. 2011). Additionally, animal studies demonstrated that a reduction in the expression of GABA_A_/_B_ receptors results in a behavioural pattern resembling anxiety (Arora et al. 2024).

Surprisingly, despite exhibiting hyperlocomotion in the open field, PSD mice were unable to maintain consistent performance on the rotarod apparatus. Hyperlocomotion is often associated with underlying mitochondrial stress or central fatigue, reflecting the physiological toll of sustained hyperactivity. Together, these behaviours highlight the dual impact of sleep deprivation on behavioural stimulation and physical endurance. The rotarod test, commonly used to evaluate motor coordination and fatigue, serves as a reliable measure of these effects. In a clinical study, 24 h of sleep deprivation was found to influence inflammatory cytokines, stress hormones, and emotional states in participants. Acute sleep deprivation increased negative emotions, such as anxiety and fatigue, highlighting its systemic effects (Thompson et al. 2022). Sleep deprivation affects not only neural functions but also induces muscular fatigue, particularly through mitochondrial dysfunction (Sharma et al. 2023). Herein, the fatigue was easily elicited by rotarod than that of open field. Notably, administration of DAPA may reduce rotarod failures by alleviating central stress through lowering stress hormones or protecting mitochondria via SIRT1 activation (Gao et al. 2024). A key debate in sleep research revolves around whether the molecular and cellular changes observed after sleep deprivation (SD) result directly from sleep loss or are mediated by stress. The multiple-platform SD method, which involves immobilization and social isolation, induces stress, as evidenced by increased corticosterone and ACTH levels in SD mice. However, this stress component is not a limitation; instead, it enhances the model’s construct validity, given that stressful life events are well-established contributors to the onset of BD (Zhang et al. 2022). Consequently, the current SD model, which integrates mild stress with sleep disturbances—particularly REM sleep deprivation—satisfies key validity criteria and provides a robust framework for studying the mechanisms underlying mania (Abrial et al. 2014).

Previous literature regarded PSD as an environmental stress model by disrupting the hypothalamus-pituitary axis (HPA). Valvassori et al., (2017), claimed that this modulation can lead to a spike in ACTH and corticosterone levels. Worthy to mention, a mutual interplay between stress hormones and GABAergic machinery was described by Mody & Maguire, (2011), where they reported that HPA is controlled by GABAergic inhibition, and contrariwise, stress hormones modulate GABA receptors’ expression. Moreover, another critical correlation was found between stress hormones and neurotrophic functions in the brain. Dal-Pont et al., (2019), linked the decrease in BDNF level to the increased ACTH hormone levels in PSD rats which is in agreement with the present results. Neurotrophic factors are crucial not only for neurogenesis and neural connectivity (Numakawa et al. 2018) but also for maintaining GABAergic synaptic transmission (Porcher et al. 2018). The trophic factor BDNF influences GABAergic transmission bidirectionally, demonstrating positive and negative effects via multiple intracellular signaling mechanisms. However, studies stated that BDNF regulates GABA uptake in hippocampal neurons by altering the levels of GABA transporting enzyme GAT-1 (Vaz et al. 2008) and potentiates the inhibitory strength of GABA_A_ receptors by controlling the Cl^−^ flux, maximizing the hyperpolarization force. In cortical neurons, the transcriptional regulation of presynaptic GAD65, which is correlated more with plasticity-related functions, is regulated by BDNF through BDNF/tropomyosin-related kinase B (TrkB) signaling pathway (Sánchez-Huertas & Rico 2011). Postsynaptically, GABA_B_ receptors’ stimulation plays a key role in BDNF release in rat hippocampus (Kolarow et al. 2007), where the latter orchestrates the cell surface expression of GABA_A_ receptors and modulates GABA_A_ trafficking between synapses and endosomes (Jovanovic et al. 2004). This was also evidenced in hippocampal tissue, where BDNF promoted GABA_A_ clustering at postsynaptic membranes (González 2014), this GABA_A_ clustering also has a positive feedback on BDNF release (Porcher et al. 2011).

A growing body of evidence has explored the effect of sleep on BDNF expression. Since REM sleep is associated with memory consolidation and learning, BDNF has been implicated in regulating sleep drive during wakefulness and sleep (Rahmani et al. 2019). Previous work reported the variation in BDNF level in the diurnal cycle, influencing neuronal plasticity and synaptic transmission. In clinical studies, SD was linked to diminished levels of BDNF accompanied by increased cortical excitability (Kuhn et al. 2016). Indeed, PSD is associated with lower levels of BDNF in rats, which has a deleterious impact on hippocampus-related functions (Alhaider et al. 2011; Zagaar et al. 2013). This suppression negatively affects the genetic expression of its downstream targets, including cAMP response-element-binding (CREB), Synapsin I and calcium–calmodulin-dependent protein kinase II (CAMKII). These downstream modulators are responsible for synaptogenesis, dendritic pruning and neurotransmitters’ release (Guzman-Marin et al. 2006). Likewise, preclinical and clinical data proclaimed that GDNF was markedly downregulated following SD (Sochal et al. 2024; Wang et al. 2024). However, results obtained from studies regarding SD impact on neurotrophic factors lack consensus. A recent study proclaimed that acute SD enhances BDNF/GDNF levels as a compensatory mechanism against stress-related disruption triggered by SD (Gorgulu et al. 2021). Moreover, Fujihara et al., (2003), stated that rats subjected to SD had surprisingly higher levels of BDNF in their hippocampus. This discrepancy is likely due to species variation and the SD strategies used differing in duration and intensity. Nonetheless, the diminished BDNF directly affects its downstream autophagic signaling pathway as well. BDNF was proven countlessly to be an upstream activator of liver kinase B1 (LKB1) and consequentially AM*P-*activated protein kinase (AMPK) (Huang & Li 2022; Kuwako & Okano 2018). Simultaneously, activation of AMPK was proven to enhance BDNF release in rodent hippocampus (Xu et al. 2013). These metabolic sensors are well-recognized for their role in macro-autophagy, energy metabolism and reactive oxygen species (ROS) control, which are impaired in most neurodegenerative disorders (Nixon 2013). The activation of some kinase complexes by AMPK mediates the catabolic activity of autophagy to shield cells under stress. This degradation process, driven by LKB1/AMPK/LC3 pathway, is consummated by autophagosome formation. While the role of BDNF/GDNF in regulating autophagy is still elusive, recent research revealed a rather interesting correlation between these trophic factors and autophagic flux. Several lines of evidence proposed an ability of BDNF to induce autophagy in hippocampal and cortical neurons (Chen et al. 2013; Sidibe et al. 2022). This effect was inferred when BDNF administration resulted in increased levels of the autophagosome indicator, microtubule-associated protein light chain 3II (LC3II), the lipidated form of LC3I. Sidibe et al., (2022) concluded that BDNF increased autophagosome density but without any co-migration between these two markers. Moreover, BDNF-treated neurons showed increased autophagosome formation via BDNF/TrkB retrograde transport, however, the effect on autophagic markers was not assessed (Kononenko et al. 2017). Interestingly, GDNF was also proven to have a modulating role in autophagic flux. Wang et al., (2024) noticed a positive regulation of autophagy following GDNF microinjection in mice forebrain. This was confirmed by a surge in LC3 I/II ratio along with a curbed p62 level, a core autophagy marker, in GDNF-treated mice. Worthy to note, p62 degradation signifies autophagy completion and maturation, reflecting the fusion between autophagosomes and lysosomes. On the other hand, studies have demonstrated that BDNF and GDNF suppress autophagic machinery as a neuronal survival mechanism against mitochondrial dysfunction (Shang et al. 2010; Smith et al. 2014; Wu et al. 2017). The rationale behind these disputes can be attributed to the variation in experimental model systems and neuronal ensembles assessed for autophagy.

In fact, several reports revealed that autophagy plays a role in axonal development (Yamaguchi et al. 2018) and dendritic pruning (Tang et al. 2014). In addition to having its share in sustaining synaptic plasticity and enhancing cognition (Fu et al. 2017). This autophagic malfunction anecdote extends far beyond accumulation of proteins and aggregates in the neurons. Defective autophagy can also lead to the accumulation of autophagosomes in neurons causing their death. Recent studies described a link between the upregulation of p62 protein and autophagic deficiency (Hui et al. 2019). This elevation in p62 level was observed in neuropsychiatric disorders and was associated with behavioural changes which is consistent with the current findings. According to Sumitomo et al., (2018), this upsurge can inhibit GABA_A_ receptor-associated protein (GABARAP) which orchestrates the surface presentation of GABA_A_ receptor on postsynaptic membranes. The interaction of p62 and GABARAP can cause an imbalance between excitatory-inhibitory machinery in the brain that may provoke manic events. In addition, Grunwald et al., (2020), discovered a positive regulation of kinase enzymes responsible for autophagosome formation by GABARAP. Moreover, Grunwald (2020) conceded that this positive interaction is suppressed by autophagy related gene 7 (ATG7) depletion. This aligns with the present results as the manic mice represented a tremendous increase in the autophagic protein LC3I, the precursor of LC3II, a stepping-stone in the autophagic pathway (Runwal et al. 2019). The over-presence of p62 and LC3I in autophagic compartments indicates deactivated autophagosome formation. This input/output relation is crucial as it implies a buildup of LC3I encountered by a curbed ATG7 and consequently, LC3II. Peremptorily, this autophagic insufficiency can hinder cellular homeostasis and neuronal viability leading to social and behavioural deficits. Not to mention, the dramatic decline in GABARAP affecting neuronal transmission.

Sleep disturbances, specifically REM sleep, have been involved in many neurodegenerative disorders. REM sleep loss, for example, shares the same characteristics of neuronal damage and aging, including disruptions in memory encoding/consolidation and cognitive skills (Beaulieu & Godbout 2000; Mander et al. 2017). Moreover, REM sleep has also been implicated in synaptic refinement and neuronal integrity. Accumulating evidence confirmed that SD provokes aberrant autophagy in multiple neuronal ensembles (Cao et al. 2019; Cheng et al. 2021; Dai et al. 2021). Research proposed that SD can diminish intracellular Ca^2+^ levels, which is critical for AMPK activation (Chauhan & Mallick 2019; Høyer-Hansen et al. 2007). As a corollary, this can interfere with autophagy signaling pathways and adversely affect mitochondrial functions, leading to apoptosis. In addition, disturbed autophagy can impair neuronal excitability by prompting GABA_A_ entrapment and overexpression of inactive inwardly-rectifying K^+^ channels in GABAergic neurons (Lieberman et al. 2020; Pigulevskiy et al. 2020). This was further validated by the reduced GABA levels found in rodents exposed to variant SD protocols (Mehta et al. 2017; Mohamed Kamal et al. 2010). Hereforth, preclinical studies suggested that induction of autophagy can aid in the neuroprotection in disorders such as Alzheimer’s and Parkinson’s (Nah et al. 2015; Uddin et al. 2018). Interestingly, DAPA ameliorated the autophagic dysfunction evoked by PSD and was able to restore neurotrophic factors’ and autophagic markers’ levels. Indeed, the multipronged effects of DAPA emphasize its role in modulating the autophagic awry in multiple neural disorders (Arab et al. 2023; Kamel et al. 2022, 2023). However, Hawley et al., (2016), opposed these results, proclaiming that AMPK was not an ideal marker for DAPA action in cell culture. This contradiction could be attributed to the fact that the study inspected DAPA effect only *in-vitro* in Hawley’s (2016) work. Altogether, in respect of PSD pathophysiology, the curtail of the GABAergic system can be ascribed to the attenuation of the autophagic clearance, the upsurge in stress hormones as well as BDNF depletion.

Early studies depicted the interrelations of proinflammatory response and cortisol production (Taishi et al. 1998). According to Frank et al., (2015), glucocorticoids possess a permissive trait that triggers neuroinflammation in case of stress or brain injury. This generation of inflammatory cytokines is engaged in a feed-forward loop with glucocorticoids (Frank et al. 2015; Kelly et al. 2018). A core stressor as SD can also trigger neuroinflammatory responses through microglia activation (Garofalo et al. 2020). Nonetheless, the disruption of autophagic flux has also been associated with sensitizing the cells to trigger neuroinflammation (Wang et al. 2015). Jin et al., (2018), described the autophagic dysfunction aggravated by TNF-α that led to neurodegeneration. This circuit was accompanied by increases in other inflammatory markers like IL-1β and anti-inflammatory ones such as IL-10. Here we found that inflammatory cytokines; TNF-α, IL-1β and IL-4 showed a robust stream after PSD induction, compensated by a surge in the anti-inflammatory marker IL-10, events that were reversed after DAPA administration. These outcomes are in harmony with other studies establishing the neuroinflammatory background behind sleep deprivation (Ashley et al. 2016; Wadhwa et al. 2018), and promoting the anti-inflammatory role of DAPA (Lee & Riella 2024; Sa-Nguanmoo et al. 2017). Taken together, these outcomes are supported by the histopathological report revealing the neuronal degeneration in PSD animals contended with the intact meninges in DAPA-treated mice. On a side note, the PSD-induced uprise in cortical and hippocampal SGLT2 expression sheds light on the implication of this transporter in triggering mania that needs to be verified in upcoming research.

This study has limitations that warrant consideration. While the paradoxical sleep deprivation (PSD)-induced mania-like model is widely used, it only partially captures the complex neurobiology of BD in humans. To enhance translational validity, future studies should incorporate chronic and genetic BD models that better reflect the disorder’s pathophysiology. Second, the present study focused exclusively on male mice, despite the sex-dependent variations in mania-like behaviour prevalence, and pharmacological response. Further research should explore DAPA influence in female models to assess potential sex-based therapeutic differences. Lastly, the short duration of DAPA treatment in the present study may not fully capture its long-term neuroplastic adaptations. Moreover, these studies should extend the treatment period to evaluate the sustained impact of DAPA on autophagic homeostasis, neurotrophic signaling, and behavioural outcomes in BD models. Finally, it would be preferred to incorporate additional stress-control groups that could help further delineate the effects of sleep deprivation versus apparatus-induced stress on molecular and behavioural outcomes.

## Conclusion

The role of autophagy in manic disorders remains complex and somewhat contradictory across studies. Therefore, investigating the impact of DAPA on autophagic flux was crucial. The findings of this study suggest that DAPA exerts its anti-manic effects through a dual mechanism: enhancing autophagic clearance and modulating GABAergic transmission, which collectively contribute to the attenuation of manic-like behaviour. Additionally, DAPA’s well-established anti-inflammatory properties further reinforce its neuroprotective potential. Histopathological analyses corroborate these findings, providing strong preclinical evidence—reported for the first time—supporting DAPA’s therapeutic role in paradoxical sleep deprivation (PSD)-induced mania in mice.

## Supplementary Information

Below is the link to the electronic supplementary material.

Supplementary file1 (JPG 892 KB)

Supplementary file2 (JPG 874 KB)

Supplementary file3 (JPG 916 KB)

Supplementary file4 (JPG 882 KB)

Supplementary file5 (JPG 740 KB)

Supplementary file6 (JPG 737 KB)

Supplementary file7 (PNG 943 KB)

Supplementary file8 (JPG 918 KB)

Supplementary file9 (JPG 981 KB)

Supplementary file10 (JPG 992 KB)

## Data Availability

All relevant data are present in the submitted manuscript, table, and figures. Any other data will be made available on request.
