# Supplementary figures and images for: Exploring Dapagliflozin’s Influence on Autophagic Flux in Mania-like Behaviour: Insights from the LKB1/AMPK/LC3 Pathway in a Mouse Model
Source: J Neuroimmune Pharmacol. 2025 May 22;20(1):57. doi: 10.1007/s11481-025-10218-1 (PMC12098488; doi:10.1007/s11481-025-10218-1)

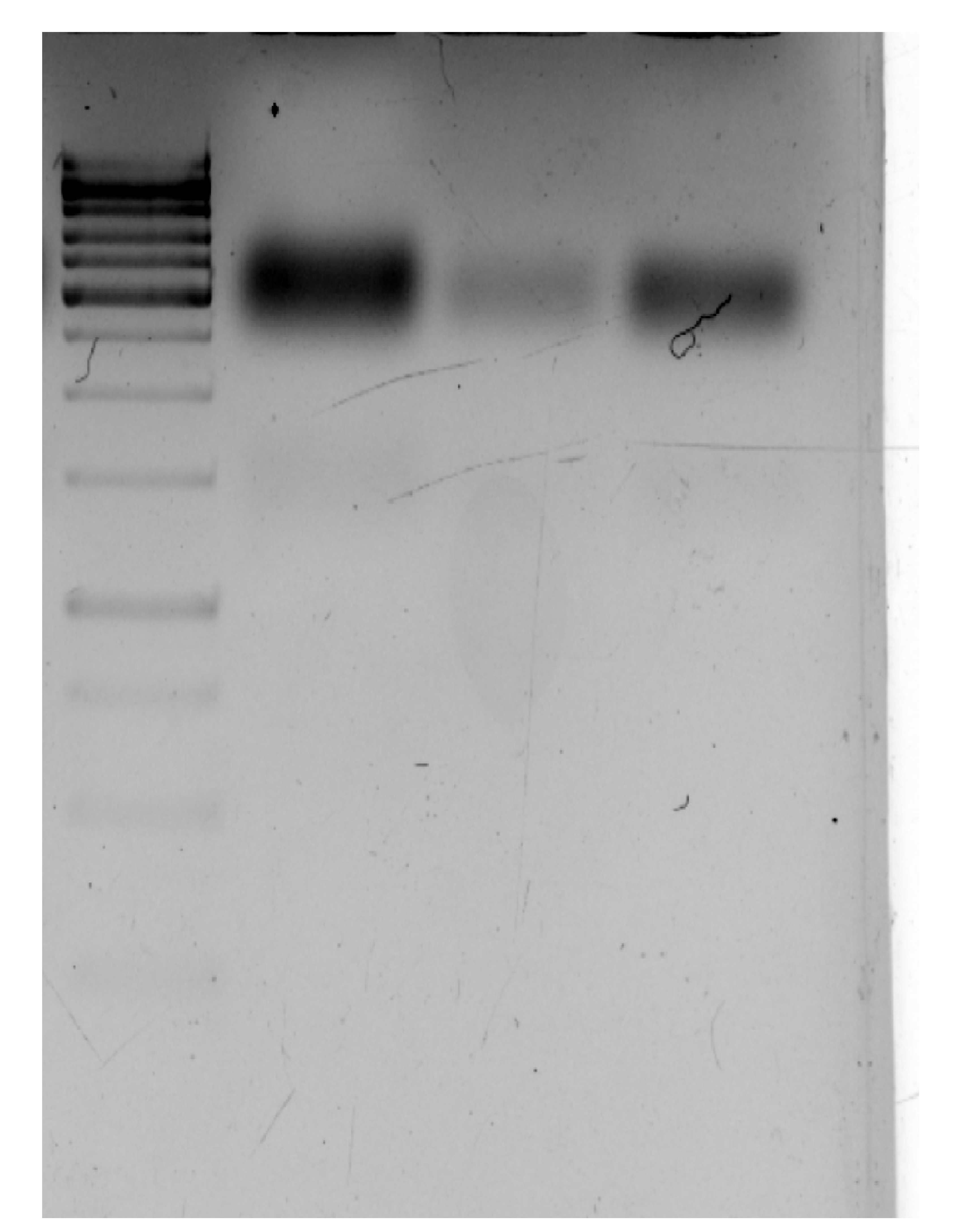

Supplement: Supplementary file 1 — Supplementary file1 (JPG 892 KB) [file 11481_2025_10218_MOESM1_ESM.jpg]

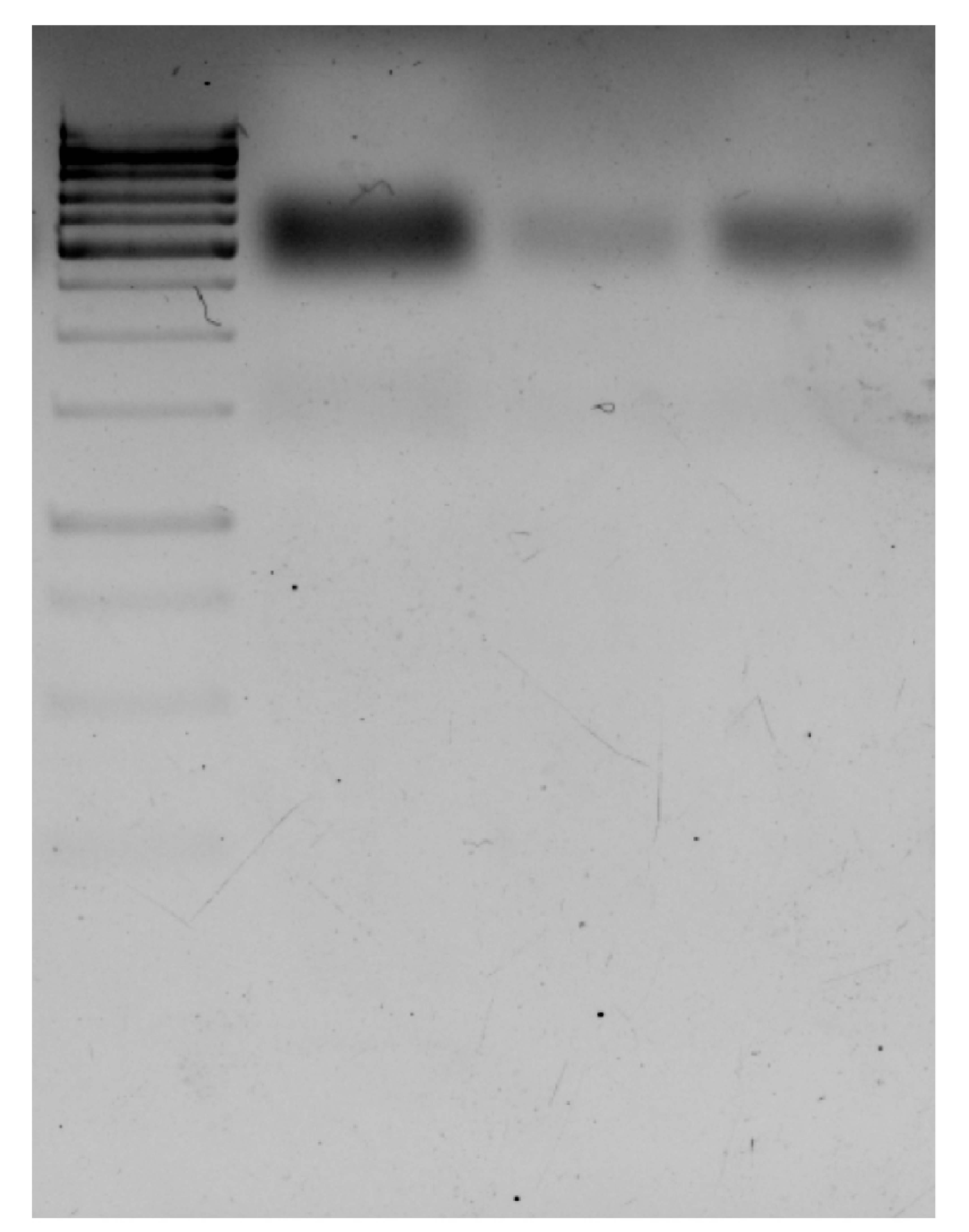

Supplement: Supplementary file 2 — Supplementary file2 (JPG 874 KB) [file 11481_2025_10218_MOESM2_ESM.jpg]

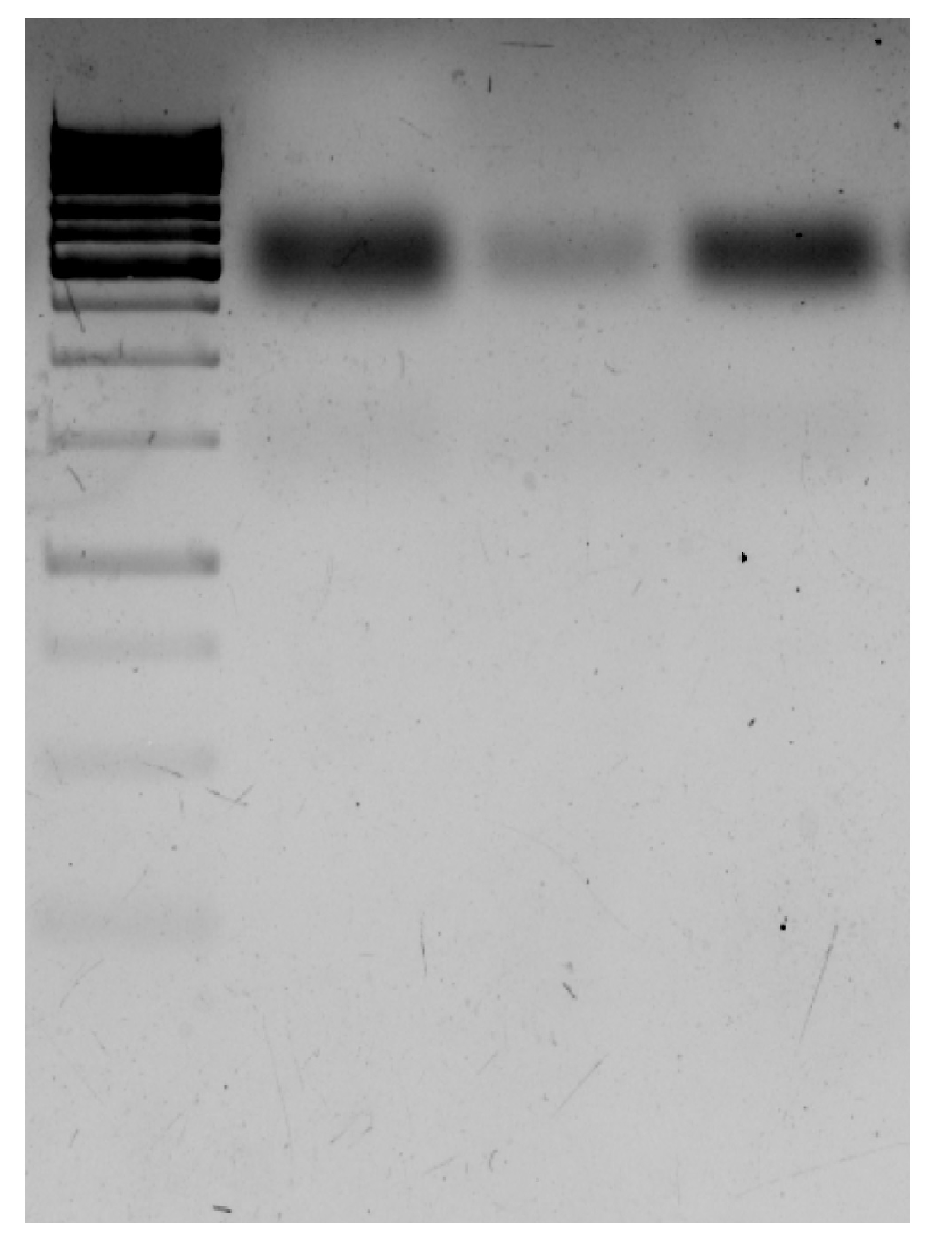

Supplement: Supplementary file 3 — Supplementary file3 (JPG 916 KB) [file 11481_2025_10218_MOESM3_ESM.jpg]

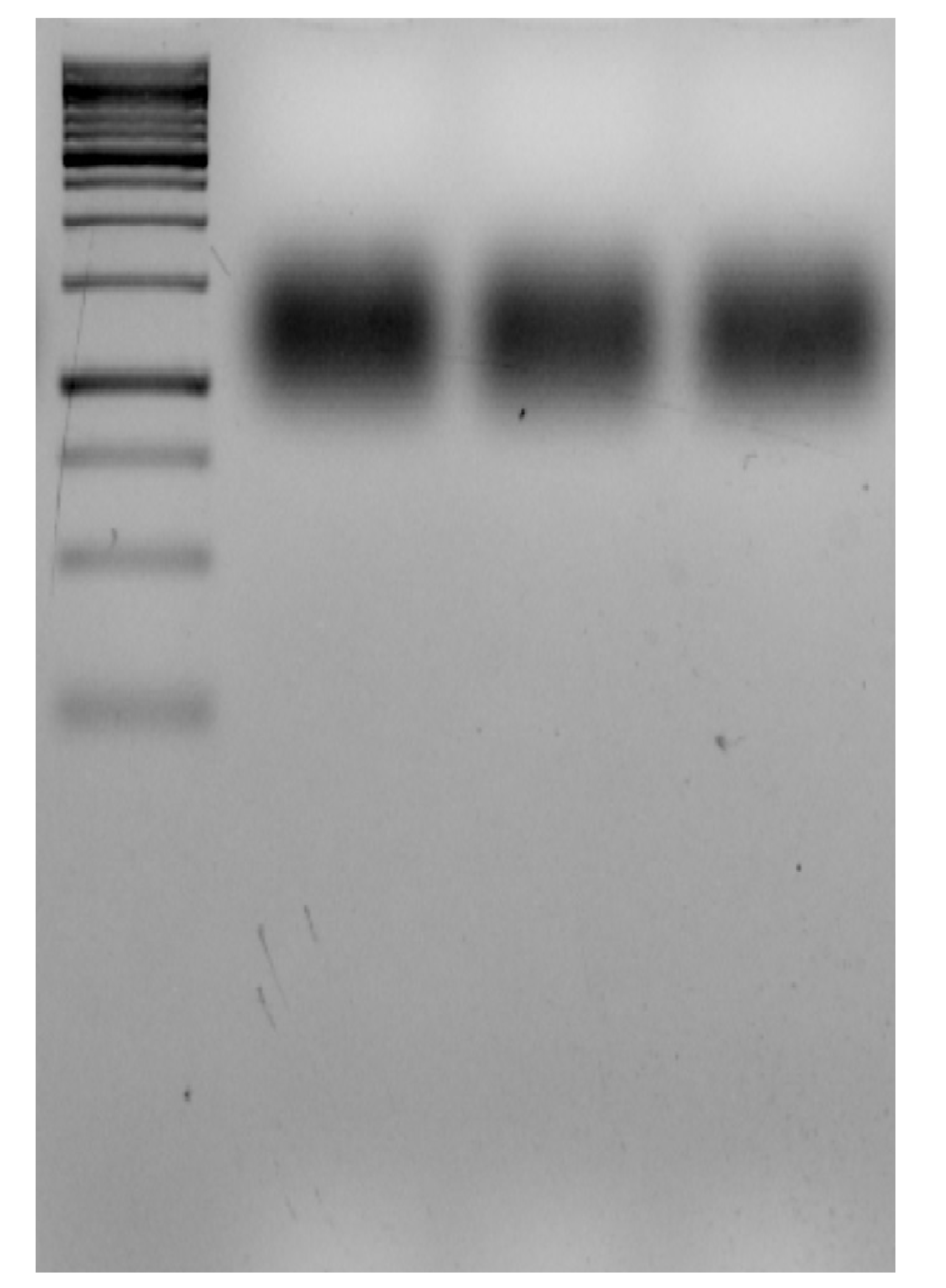

Supplement: Supplementary file 4 — Supplementary file4 (JPG 882 KB) [file 11481_2025_10218_MOESM4_ESM.jpg]

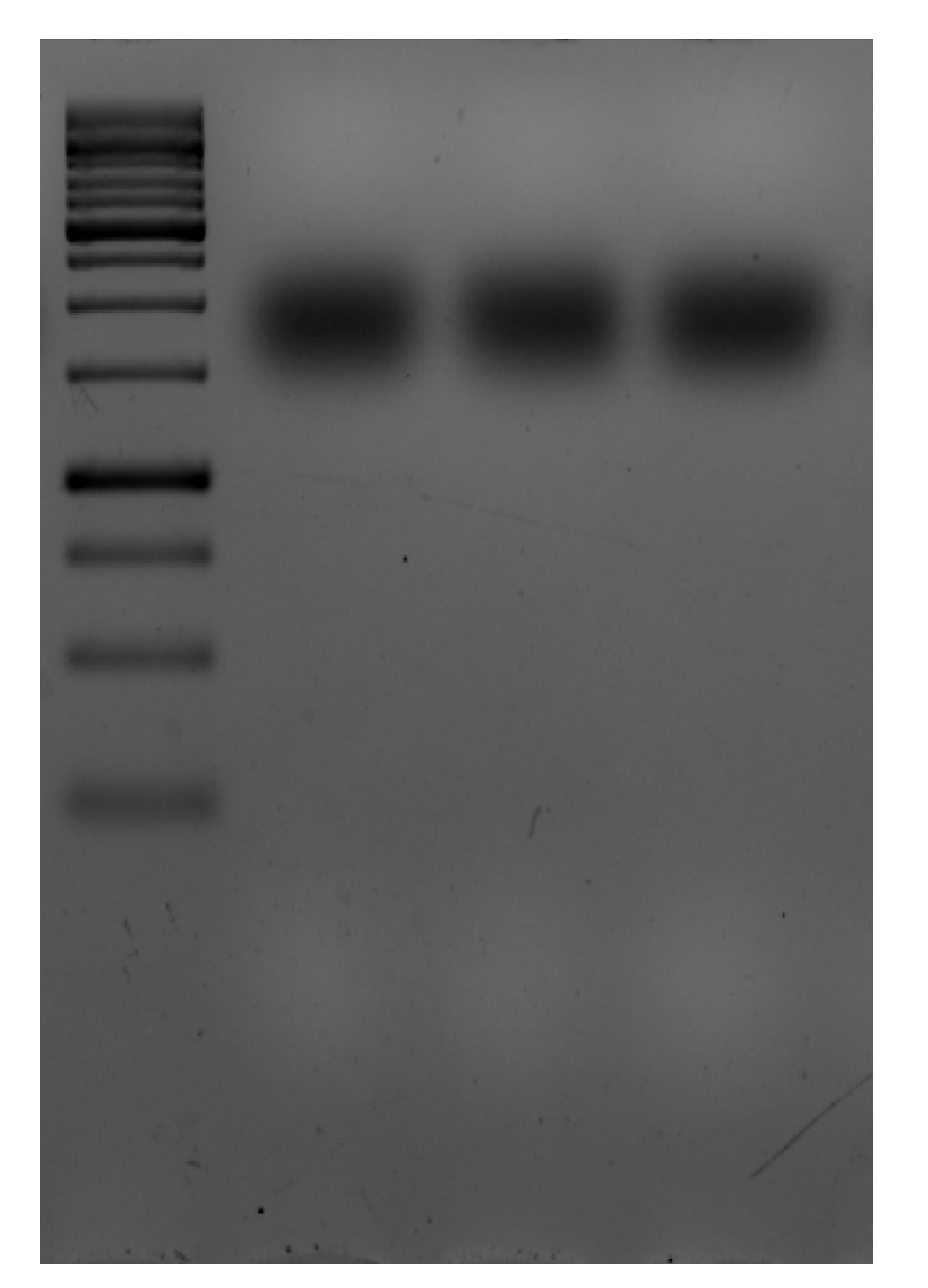

Supplement: Supplementary file 5 — Supplementary file5 (JPG 740 KB) [file 11481_2025_10218_MOESM5_ESM.jpg]

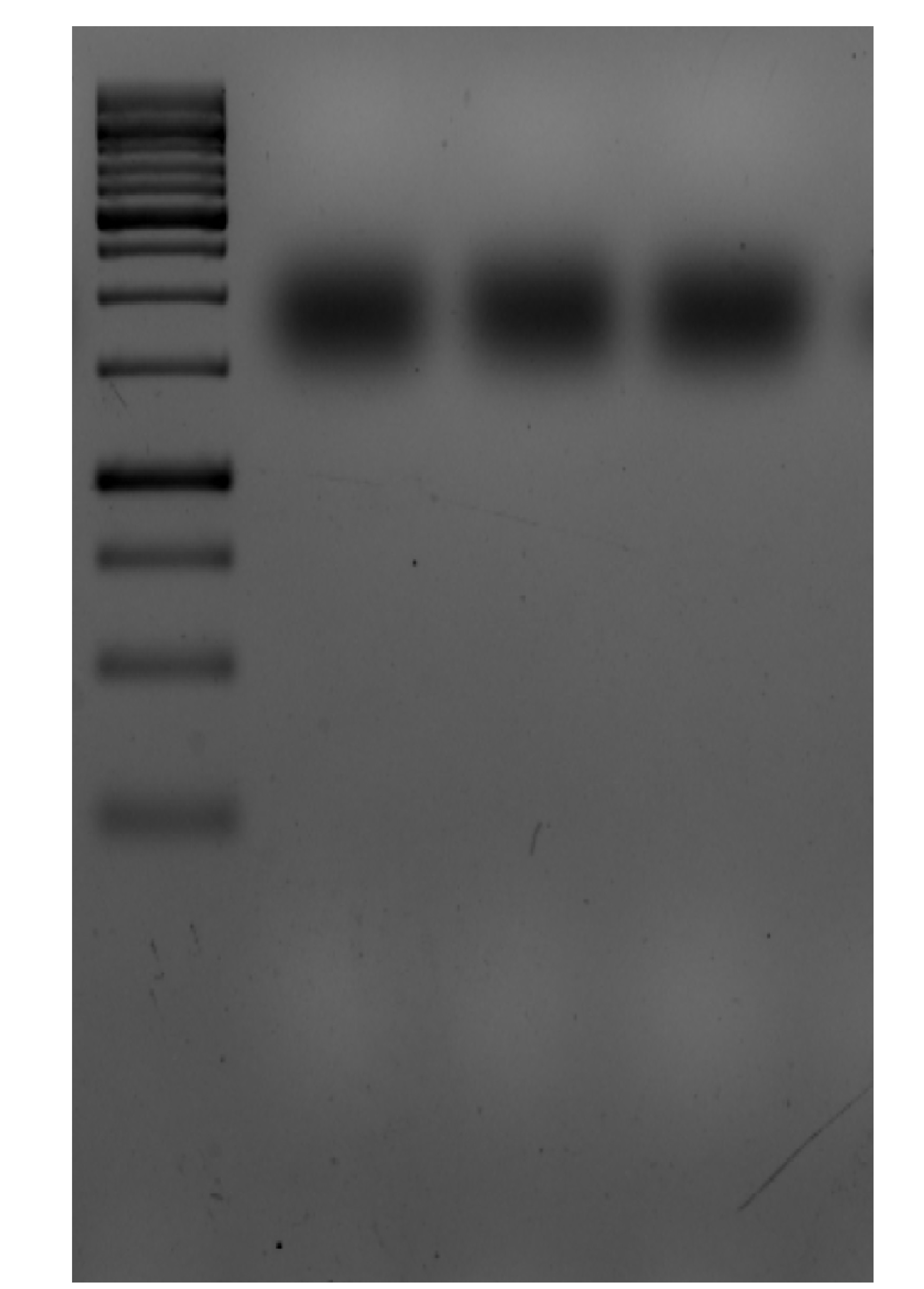

Supplement: Supplementary file 6 — Supplementary file6 (JPG 737 KB) [file 11481_2025_10218_MOESM6_ESM.jpg]

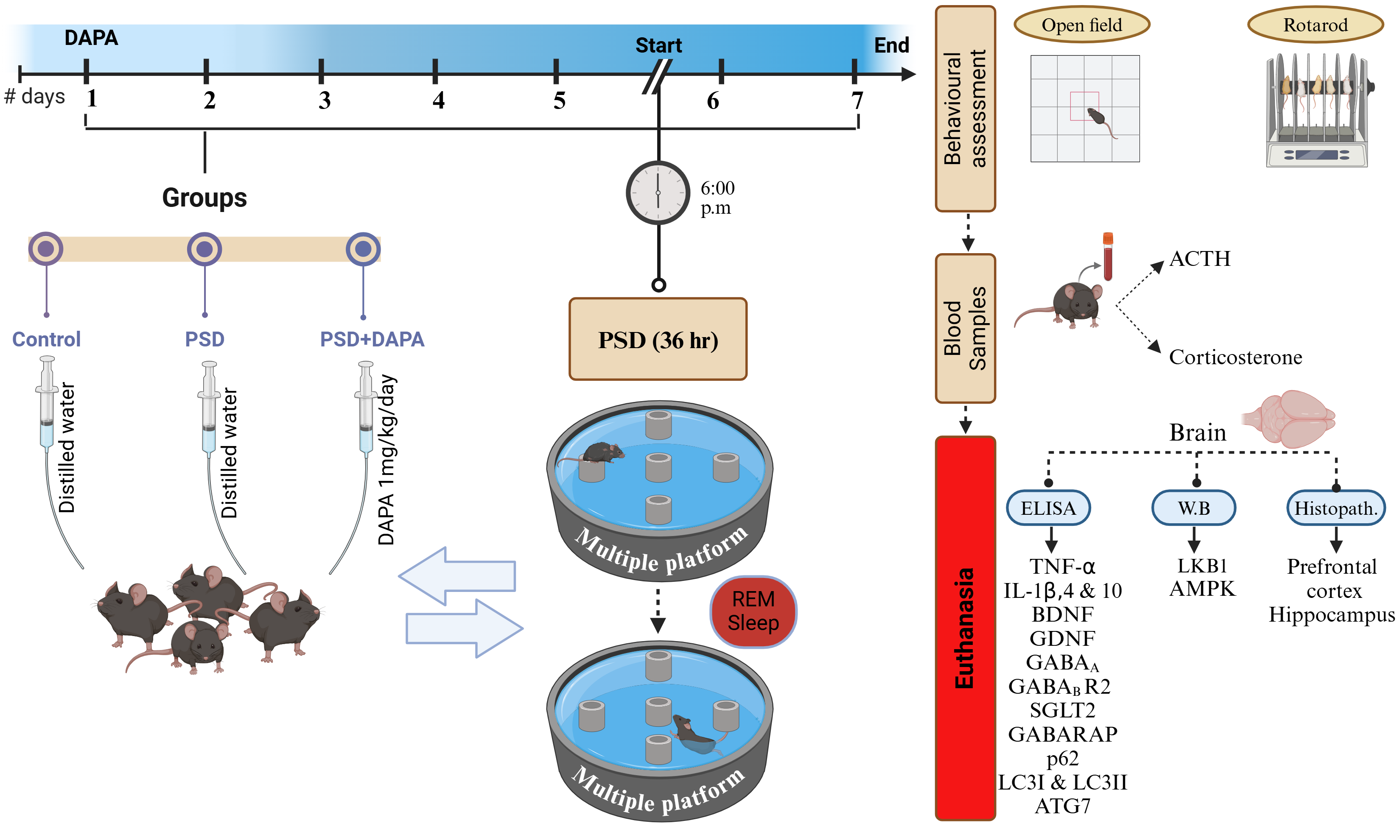

Supplement: Supplementary file 7 — Supplementary file7 (PNG 943 KB) [file 11481_2025_10218_MOESM7_ESM.png]

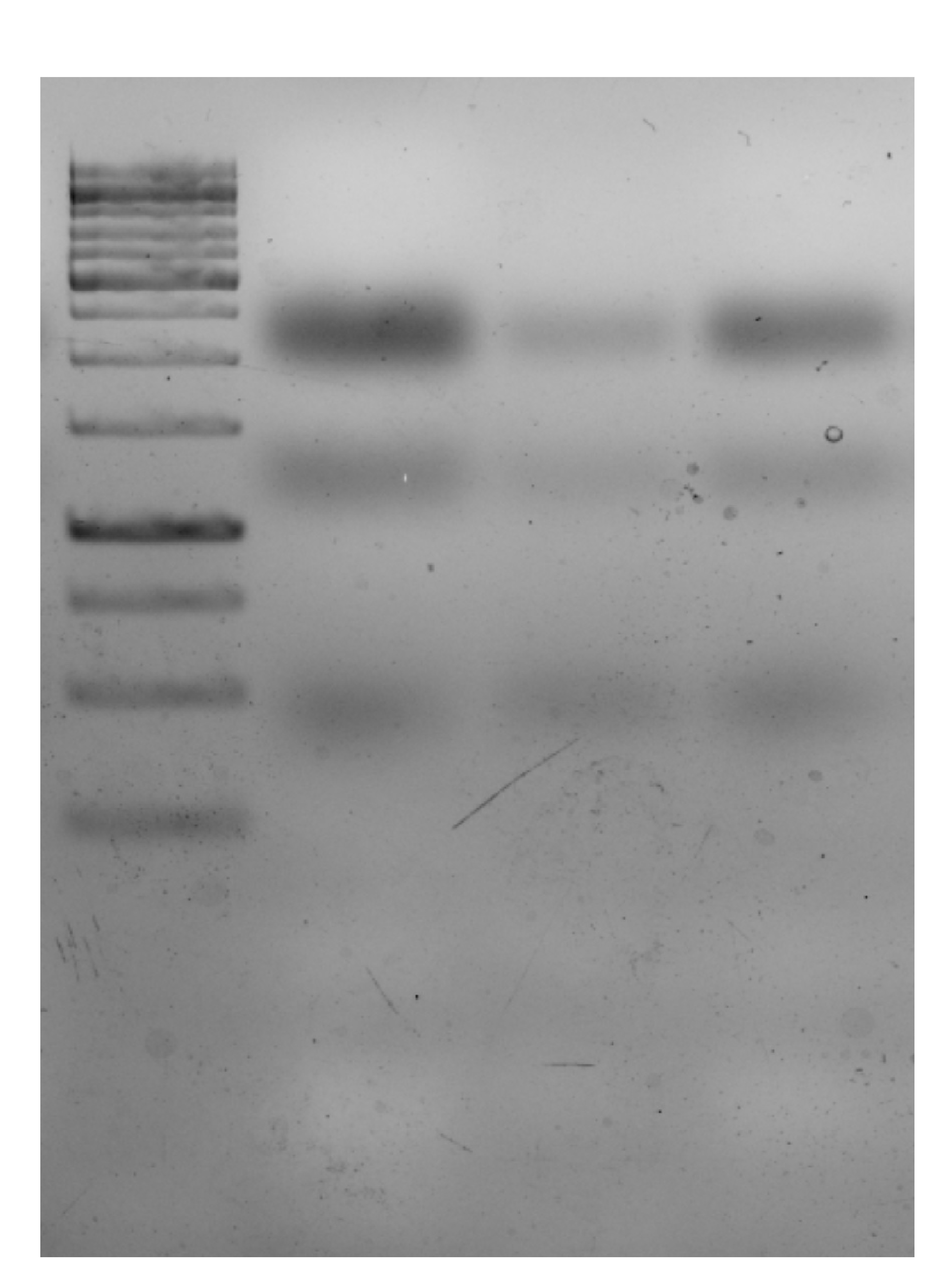

Supplement: Supplementary file 8 — Supplementary file8 (JPG 918 KB) [file 11481_2025_10218_MOESM8_ESM.jpg]

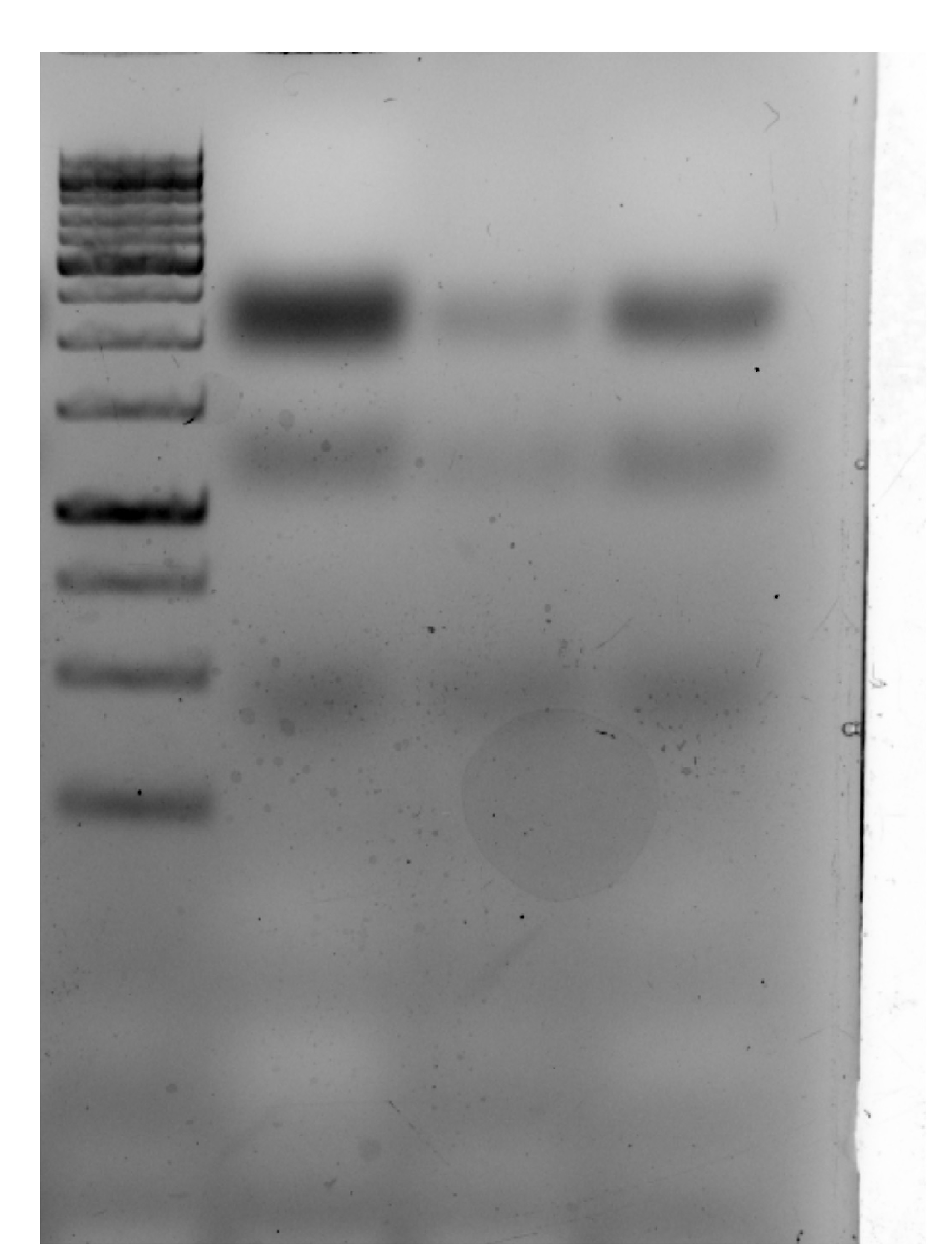

Supplement: Supplementary file 9 — Supplementary file9 (JPG 981 KB) [file 11481_2025_10218_MOESM9_ESM.jpg]

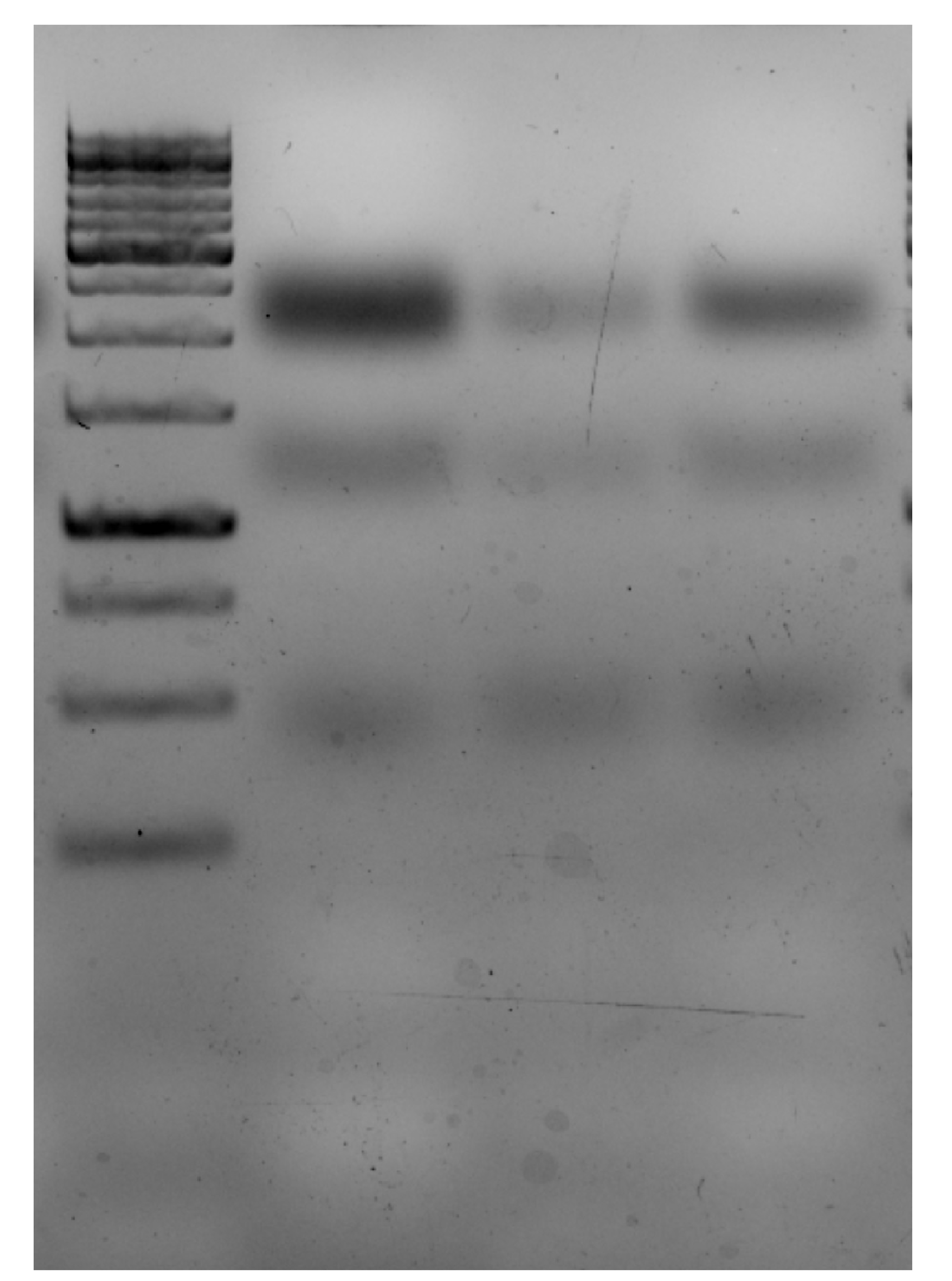

Supplement: Supplementary file 10 — Supplementary file10 (JPG 992 KB) [file 11481_2025_10218_MOESM10_ESM.jpg]
